# Discrimination in medical settings as perceived by forcibly displaced people within low- and middle-income countries: a scoping literature review

**DOI:** 10.3389/phrs.2026.1609595

**Published:** 2026-10-05

**Authors:** Kerstin M. Grosheintz-Laval, Patrick Kwizera, Mirko S. Winkler

**Affiliations:** 1 Swiss Tropical and Public Health Institute (Swiss TPH), Basel, Switzerland; 2 University Basel, Basel, Switzerland

**Keywords:** healthcare, low- and middle-income countries, migrant, perceived discrimination, refugee

## Abstract

**Objectives:**

Forcibly displaced people are among the most vulnerable populations seeking care. Understanding barriers such as perceived discrimination is critical to ensuring access to high-quality care. This review aims to provide an overview of scientific literature on discrimination faced by forcibly displaced people in healthcare settings in low- and middle-income countries (LMICs).

**Methods:**

We searched PubMed and Web of Science for peer-reviewed original studies published between March 2010 and March 2025. Screening, data extraction and reporting followed the PRISMA guidelines for scoping reviews.

**Results:**

Thirty-one studies met the inclusion criteria. Most (n = 26) applied qualitative methods. Quantitative findings showed that 10%–44% of refugees reported discrimination in healthcare. Qualitative studies described abuse, disparities in care and costs, and negative impacts on mental health and health-seeking behavior related to perceived discrimination.

**Conclusion:**

The review revealed significant gaps in research on discrimination against forcibly displaced people in LMICs, emphasizing the urgent need for more robust evidence to inform policymakers. Existing research demonstrates the importance of strengthening health systems, intercultural competence and inclusive policies to ensure respectful, high-quality care for forcibly displaced populations and other migrants.

## Introduction

The UN Refugee Agency (UNHCR) estimates that at the end of 2024, the number of people forced to flee persecution, conflict, violence, human rights violations and events seriously disturbing public order exceeded 120 million [1]. This figure includes both internally displaced people, meaning those who remained within their own country, and people who have crossed borders and fled to another country (refugees and asylum-seekers). With over 73 million people affected, internally displaced people account for the majority of the forcibly displaced [2]. Meanwhile, the refugee population has more than doubled over the past decade, rising above 42 million, with an additional 8 million people awaiting a decision on their asylum request [1, 3]. Hence, a growing share of people is living in contexts that are prone to discrimination and its health consequences.

In the first part of the *Lancet* Series on Racism, Xenophobia, Discrimination and Health, Devakumar et al. [4] define discrimination as differential and unfavorable treatment or outcome of individuals or groups based on characteristics such as race, gender, age or sexual orientation. They distinguish discrimination from other phenomena such as racism or xenophobia. While racism is an organized system, favoring certain people over others according to racial categories, which are based on actual or perceived physical appearance and ancestry, xenophobia refers to the hatred, discrimination or fear of people who are considered to be foreigners [4, 5]. The authors further explain that the root of all forms of discrimination lies in systems that classify, marginalize and oppress. In the same *Lancet* Series, Selvarajah et al. demonstrate that racism, xenophobia and discrimination are therefore important social determinants of health that have a fundamental impact on health outcomes of minoritized people [6].

Moreover, discrimination, racism and xenophobia adversely affect all key elements for receiving adequate care (availability, acceptability, accessibility and quality of care) [6, 7]. They are not only adversely related to both physical and mental health outcomes, but ultimately lead to increased mortality and reduced life expectancy [6, 8]. Several studies have shown that perceived discrimination in medical settings affects health-seeking behaviors and health outcomes. For example, the expectation of discrimination may delay access to health services or discourage people from seeking healthcare [8–12]. Furthermore, being discriminated against can be a reason for early self-discharge from the hospital or lower medication adherence [8, 13, 14]. Since forcibly displaced people belong to the most vulnerable among all patients seeking healthcare [15], understanding and evaluating potential barriers to healthcare in their resettlement location, including perceived discrimination, is essential to ensure access to high-quality care for all.

In 2023, Patillo et al. published a scoping review on racial and ethnic discrimination against racialized migrants in healthcare in various high-income-countries (HICs) across Europe [16]. No such review was found on low- and middle-income countries (LMICs), although the majority of all forcibly displaced people, including 75% of (international) refugees, are hosted by LMICs [1]. The current scoping literature review therefore aims to complement the work of Patillo et al. by mapping the current state of peer-reviewed English literature on discrimination in medical settings as perceived by forcibly displaced people in LMICs. The leading research questions were: (i) To what extent has perceived discrimination experienced by refugees and internally displaced people been investigated in medical settings in LMICs? (ii) What were the research methods used? and (iii) What are the remaining knowledge gaps?

## Methods

The methodology of the review was inspired by the approach of Arksey & O’Malley and the recommendations on the framework of Levac et. al [17, 18]. Therefore, the review was carried out according to the following stages: (i) identification of relevant studies; (ii) selection of studies; (iii) extraction of data; and (iv) summary and report of results. Reporting was conducted according to the “Preferred Reporting Items for Systematic reviews and Meta-Analyses extension for Scoping Reviews (PRISMA-ScR): Checklist and Explanation” [19].

### Search terms and strategy

Relevant peer-reviewed studies published between 11 March 2010 and 10 March 2025 were identified in PubMed and Web of Science. The search string consisted of four blocks around the following themes: (i) refugee; (ii) healthcare; (iii) discrimination; and (iv) LMICs. The search string was tailored to the specific features of the two databases, with Medical Subject Headings (MeSH) terms applied in PubMed to expand the search (the complete search strings for the individual databases are provided in the review protocol in Supplementary Material 1 – Scoping Literature Review Protocol and Methods). Three previously identified studies on the topic were used to validate and adapt the search terminology [20–22]. All hits with an English abstract were included, irrespective of the language of the full text. LMICs were defined according to the World Bank (“World Bank Country and Lending Groups”, March 2025 [23]).

### Literature screening

Titles and abstracts were screened in parallel by K.G. and P.K. using EndNote (version 21.5, Clarivate Analytics, United States) for data management and Covidence (2025 Covidence, Veritas Health Innovation, Australia) to identify relevant studies. After screening the first 100 studies, the inclusion and exclusion criteria were refined through discussion with input from M.S.W. and subsequently applied consistently. Disagreements were resolved by consensus between K.G. and P.K., with unresolved cases discussed with M.S.W. Duplicates were removed automatically by Covidence and additionally through manual checking. Full-text screening was conducted by K.G., with final inclusion decision discussed with P.K. Studies were considered eligible if they (i) included data on discrimination in medical settings as perceived by forcibly displaced people in LMICs; (ii) aimed to investigate discrimination in healthcare or barriers to healthcare access; and (iii) had full text available in English within the University of Basel’s access rights. All forcibly displaced people, regardless of age, were considered. Studies were excluded if populations were not clearly defined as forcibly displaced, if results were not reported separately from other migrant or non-migrant groups, if discrimination was reported by non-migrants (e.g., healthcare worker’s perspectives), or if conducted in HICs. All peer-reviewed studies with primary data were included, irrespective of methodology, author’s origin, or data time frame studied. Table 1 summarizes in- and exclusion criteria.

**TABLE 1:** Overview of inclusion and exclusion criteria (Basel, Switzerland, 2025).

Inclusion and exclusion criteria	
Inclusion criteria	• Data on discrimination in medical settings as perceived by forcibly displaced people AND • Aim of study to investigate discrimination in healthcare AND/OR exploring barriers to healthcare access/inequalities in healthcare AND • Full text retrievable in English with the access rights of the University of Basel
Exclusion criteria	• Described discrimination not from the perspective of the migrant • Results not primary data • Literature not peer-reviewed • Grey literature (e.g., comments, articles, policies) • Unclear or non-migrant study population, voluntary migration (e.g., labor migration) • Data exclusively focusing on HICs or not possible to extract results for LMICs independently • Full text paper not retrievable in English within the access rights of the University of Basel

### Data analysis

Data from studies included in the full-text was extracted by K.G, using a data charting form developed in Microsoft Excel (version 16.98, Microsoft Corporation, United States) with input from M.S.W. Extracted variables included: title, first author, host countries studied, journal of publication, year of publication, study population, number of participants, age of population, type of study and methods (used for analysis of discrimination), aim of the study, main findings, conclusion and main recommendations. Data analysis and the generation of tables and figures were conducted in Excel. The PRISMA flow diagram was generated using Covidence. A descriptive approach was used to summarize studies by publication year, geographical scope, study population, methodology and objectives. Key findings and recommendations were grouped into subthemes using inductive coding. Detailed study characteristics and results are presented in Supplementary Material 2 – Table of Included Studies (Objectives and Methods) and Supplementary Material 3 – Table of Included Studies (Findings and Recommendations). The manuscript was written by K.G. using Microsoft Word (version 16.102.1, Microsoft Corporation, United States). ChatGPT (version GPT-4o-mini, OpenAI, United States) was used solely to summarize or shorten text and not for data analysis or manuscript writing.

### Ethical considerations

Since a systematic review does not involve human subjects, human tissues or animal participants, no ethics committee approval was required.

## Results

### Overview of included studies

Through the initial search in PubMed and Web of Science a total of 2463 papers were identified. After removing 455 duplicates, 2008 studies were screened by title and abstract, of which 1875 were excluded. Full texts of 133 studies were assessed, leading to the exclusion of 103 papers due to incorrect outcomes (n = 47), study population (n = 36), objectives (n = 11), or host country (n = 3); one study lacked primary data. Two studies could not be retrieved in full text and three were excluded due to non-English full texts. This left 30 studies for inclusion. One additional study was identified through reference screening, bringing the total to 31 studies included in this scoping literature review (Figure 1).

**FIGURE 1:**
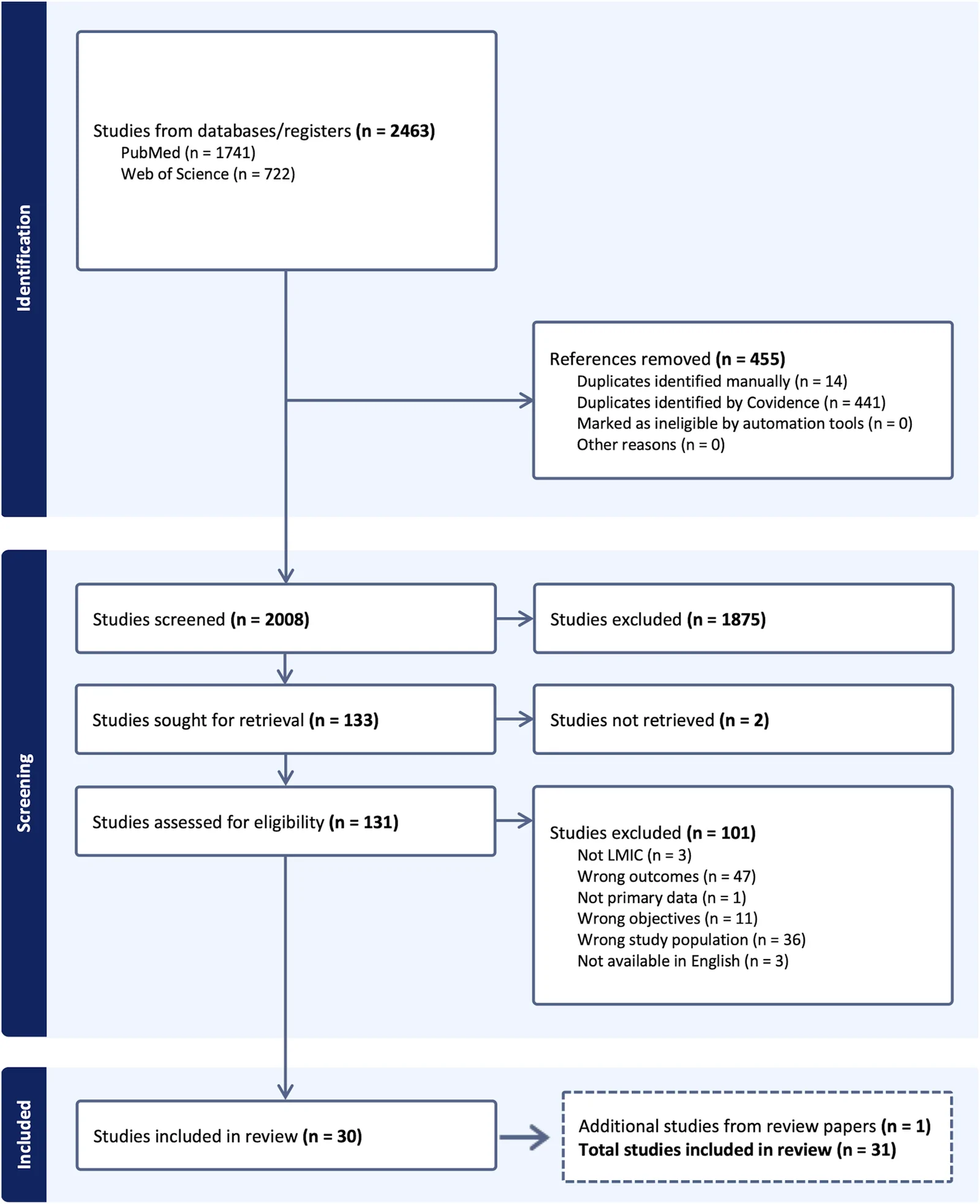
Flow diagram showing the number of studies identified in PubMed and Web of Science, screened and included in the scoping literature review (Basel, Switzerland, 2025).

### Study characteristics

#### Year of publication

Most studies were carried out during or after the COVID-19 pandemic, with 6 and 7 studies in 2023 and 2024, respectively.

#### Geographical scope

The 31 studies included in this review were carried out in the following countries: South Africa (n = 8), Lebanon (n = 5), Colombia (n = 3), Ecuador (n = 3), Peru (n = 3), Türkiye (n = 3), Uganda (n = 3), Brazil (n = 2), Iran (n = 2), Jordan (n = 2), Kenya (n = 2), Nepal (n = 1), Pakistan (n = 1), Philippines (n = 1) and Rwanda (n = 1) (Figure 2A). (Countries are listed by their official short form according to the United Nations database).

**FIGURE 2:**
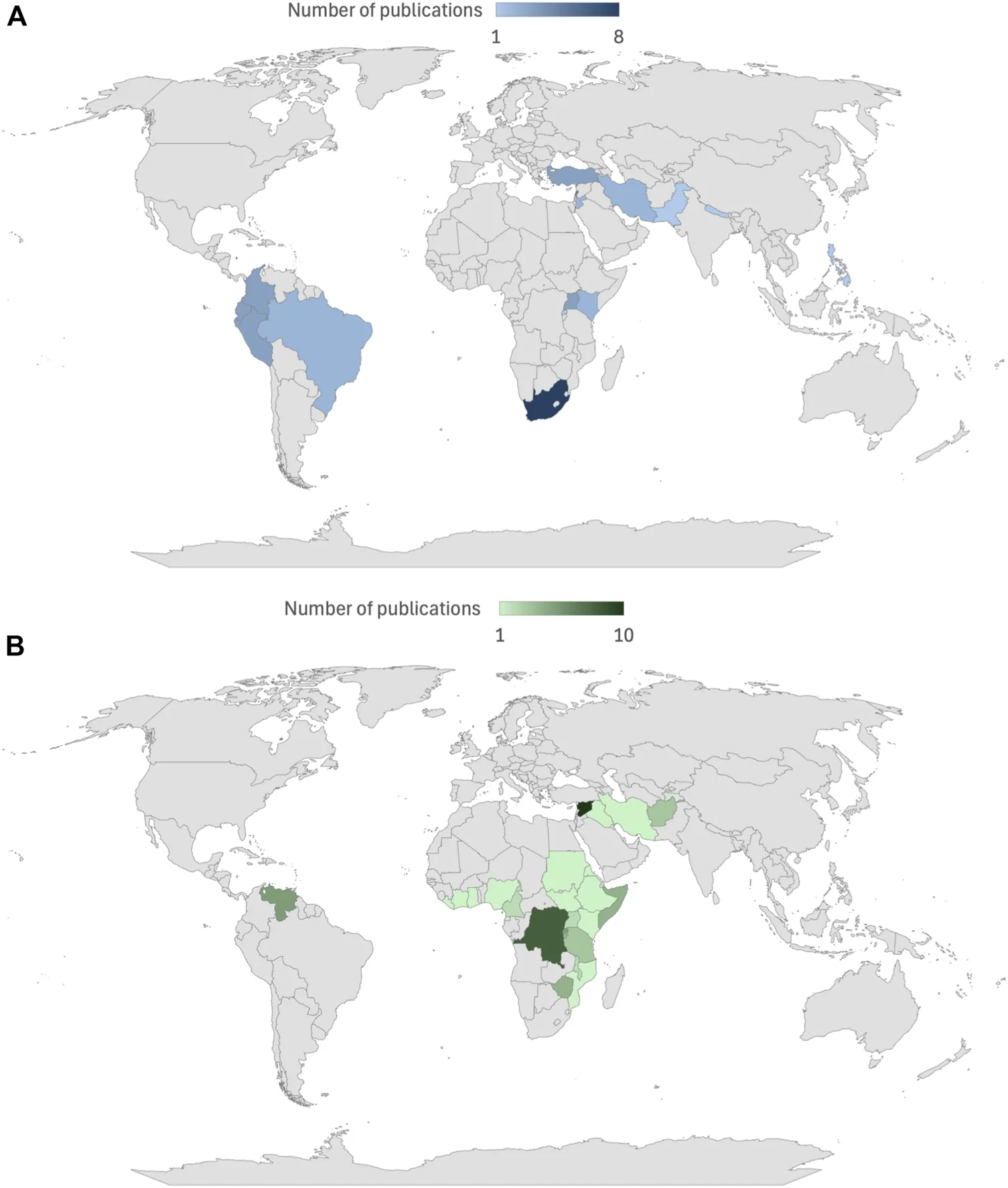
Geographical distribution and number of included studies by studied host country (panel **(A)**) and country of origin of the studied refugee population (panel **(B)**) (Basel, Switzerland, 2025).

#### Study population

The most commonly studied population were refugees from the Syrian Arab Republic (n = 10), followed by refugees from the Democratic Republic of Congo (n = 8), Rwanda (n = 5), Venezuela (n = 5), Burundi (n = 4), Somalia (n = 4), Zimbabwe (n = 4), Afghanistan (n = 3), the United Republic of Tanzania (n = 3), Cameroon (n = 2), Malawi (n = 2) and Uganda (n = 2). Forcibly displaced people from the following countries were only represented once in the included studies: Côte d’Ivoire, Eritrea, Ethiopia, Ghana, Iran, Iraq, Kenya, Libera, Mozambique, Nigeria, the State of Palestine, South Sudan and Sudan (Figure 2B). Some studies included other participants, e.g., healthcare workers or key informants of refugee organizations. In this review, only results on forcibly displaced people are shown.

While most studies focused on adults exclusively (n = 20), five studies included adolescents and adults, and two studies mentioned including all ages. Of four studies, the age of the studied population is unknown to the reader. One third of the studies (n = 11) included women only; in 9 of these studies, the respective objectives were to explore barriers to access of sexual and reproductive healthcare, especially prenatal and maternal care [20, 24–33].

#### Study design and type of data collection

The vast majority (n = 26) of the included studies used qualitative methods to investigate discrimination against refugees in healthcare. Main type of data collection was through interviews (n = 13), followed by focus group discussions (n = 7). The methods shown here are the ones from which results on discrimination could be retrieved.

#### Research objectives

Only four studies had discrimination against internationally displaced people in healthcare as their single main objective. Three studies were conducted in South Africa; two of them used qualitative methods to investigate experienced medical xenophobia among displaced people from Zimbabwe or the Democratic Republic of Congo. In contrast, the third study reported quantitative findings on disparities in prenatal care between African refugee women and women from South Africa [20, 34, 35]. The fourth study was carried out in Lebanon, applying a qualitative approach to investigate Palestinian, Iraqi and Syrian refugee women’s experiences to investigate disrespect and mistreatment during childbirth in hospitals in Lebanon [26]. All studies with details on their respective objectives, as described by the authors, are included in Supplementary Material 2 – Table of Included Studies (Objectives and Methods).

### Experiences of discrimination in medical settings

#### Quantitative results

Several studies reported on discrimination against refugee women in maternal and preventive healthcare. In South Africa, Kibiribiri et al. found that 39.2% of African refugee women would not return to the same facility if pregnant again due to staff attitudes and neglect; 43.6% perceived xenophobia, 42.3% reported carelessness, and 37.2% experienced communication barriers [20]. Similarly, Rustad et al. concluded that Sudanese refugee mothers in Uganda were less satisfied with antenatal care than host populations, with the risk of perceived discrimination rising from 4% among host women to 10% among refugees, attributed mainly to refugee status or language barriers [30]. In Colombia, Unternaher et al. found that 12%–15% of displaced Venezuelan women reported experiencing or witnessing discrimination during prenatal care visits, which was associated with a 42.8% lower likelihood of completing at least four prenatal care visits [31]. In Jordan, Ay et al. found that among Non-camp Syrian Refugees, 40.8% reported discrimination by health personnel as a barrier to health access, and 29.1% expressed distrust in healthcare providers [36]. And lastly, Benavente et al. also found that 13.9% of vaccinated refugees across various countries reported perceived discriminatory treatment during the COVID-19 vaccination process [37].

#### Qualitative findings

Numerous studies reported discriminatory attitudes, mistreatment, verbal abuse and disrespectful treatment of displaced people by healthcare providers [20, 24, 26–28, 31, 33–35, 37–45]. Refugees described being yelled at, treated as though they were stupid, denied information, blamed for expressing pain, questioned about their right to bear children in the host country, threatened, accused of burdening the health system or told to go back to their country of origin [20, 26–28, 31, 33–35, 38–45]. In some cases, physical abuse by healthcare providers was reported [26, 34, 38, 39]. Discrimination was also perceived in the tone and wording of questions about nationality [24].

Several studies described perceived discrimination in healthcare quality and cost of care [20, 27–35, 38–41, 43, 45–50]. These narratives included longer waiting hours, exclusion from services, neglect, misdiagnosis, inadequate pain management and overall treatment and higher healthcare costs based on the country of origin.

Perceived discrimination was frequently identified as a barrier to health access, leading refugees to delay or avoid seeking care and to disengage from specific facilities [20, 27, 34, 39, 40, 42, 50, 51]. Experiences of stigma and mistreatment contributed to mistrust and confidentiality concerns [36, 52, 53]. Refugees also expressed feelings of worthlessness, heightened stress and trauma, and a compromise of emotional wellbeing [33, 35, 46].

### Addressing discrimination in healthcare

Four themes emerged from the literature on improving healthcare access and reducing discrimination against displaced people in medical settings.

#### Enhance intercultural competencies in healthcare providers

Healthcare providers need to be trained to recognize the specific needs and barriers faced by refugees [28, 49]. Education in cultural diversity, antidiscrimination strategies, and legal healthcare entitlements can enhance intercultural sensitivity, empathy, and quality of and access to healthcare [20, 24, 27, 28, 33, 35, 41, 43–47, 50]. Integrating traditional treatment practices from migrants’ culture may further enhance treatment acceptance and perceived adequacy [53].

#### Strengthen health systems

Equitable care requires resilient health systems with adequate staffing, infrastructure, information systems, and supply chains [26, 29, 32, 36, 38, 40]. The use of interpreters, trained refugee healthcare workers, and telemedicine can reduce miscommunication, build trust, and improve access to care [20, 28, 29, 45]. Health services should also address specific medical and psychosocial needs of vulnerable groups, including pregnant women, displaced people with disabilities, and those with mental or chronic illnesses [29, 32, 33, 44, 52, 53].

#### Develop and promote inclusive policies

Global health commitments must be translated into national and local polices that support Universal Health Coverage [26, 41]. Inclusive policies should remove legal barriers, ensure access to healthcare regardless of legal status, and protect migrants from violence and deportation [24, 27, 30, 33, 38, 47, 48, 53]. Migrant-sensitive policies and targeted communication strategies can reduce xenophobia and improve awareness of healthcare entitlements among both refugees and healthcare staff [31, 42, 43, 45, 50, 52]. Financial barriers may be mitigated through insurance inclusion, subsidies or cash transfer programs, alongside strengthened compliance with anti-corruption policies [24, 29, 39, 43, 44, 51]. Ongoing monitoring and refugees participation in policy development are critical for responsive health systems [27].

#### Conduct further research on specific needs of refugees

Several studies identified the need for further research on refugees’ health needs, barriers to care and experiences of discrimination [20, 24, 25, 30, 32, 34, 37, 38, 44, 50, 51]. This includes examining healthcare provider’s perspectives on behaviors perceived as discriminatory, evaluating refugee programs, identifying strategies to reach underserved populations, and using more nuanced indicators to allow a distinction between objective and subjective quality healthcare [30, 34, 38, 51]. Addressing the structural drivers of marginalization and violence remains critical [25].

## Discussion

To our knowledge, this is the first scoping literature review mapping out the current state of peer-reviewed literature on discrimination in medical settings as perceived by forcibly displaced people in LMICs. Although discrimination in healthcare is a well-known phenomenon that acts as a barrier to accessing health services and may lead to worse health outcomes in several ways [6, 16, 54–59], our review shows that discrimination in healthcare, as perceived by refugees in LMICs, is a widely under-researched topic.

### Geographical scope and gaps in literature

Out of the countries listed at least once by UNHCR among the top 10 receiving countries of forcibly displaced people from 2010 to 2024 [60], only Colombia, Iran, Jordan, Pakistan and Türkiye had studies that could be included in this review. No studies were found that focused on Afghanistan, the Democratic Republic of Congo, Ethiopia, Iraq, Nigeria, the State of Palestine, Somalia, South Sudan, Sudan, the Syrian Arab Republic, Ukraine or Yemen, leaving those major refugee-hosting countries unrepresented (Figure 3A). Out of the countries listed at least once by UNHCR among the top 10 countries of origin with the most forcibly displaced people from 2010 to 2024 [60], some studies on discrimination in medical settings were done on people originating from Afghanistan, the Democratic Republic of Congo, Somalia, the Syrian Arab Republic and Venezuela. Only one study each included refugees from Ethiopia, Iraq, Nigeria, the State of Palestine, South Sudan and Sudan, and no studies including forcibly displaced people from the Central African Republic, Colombia, Myanmar, Pakistan, Türkiye, Ukraine or Yemen matching our inclusion criteria were found (Figure 3B). Hence, the significant underrepresentation of many of the most affected host countries and displaced populations identified in this scoping literature review calls for further investigation in future studies.

**FIGURE 3:**
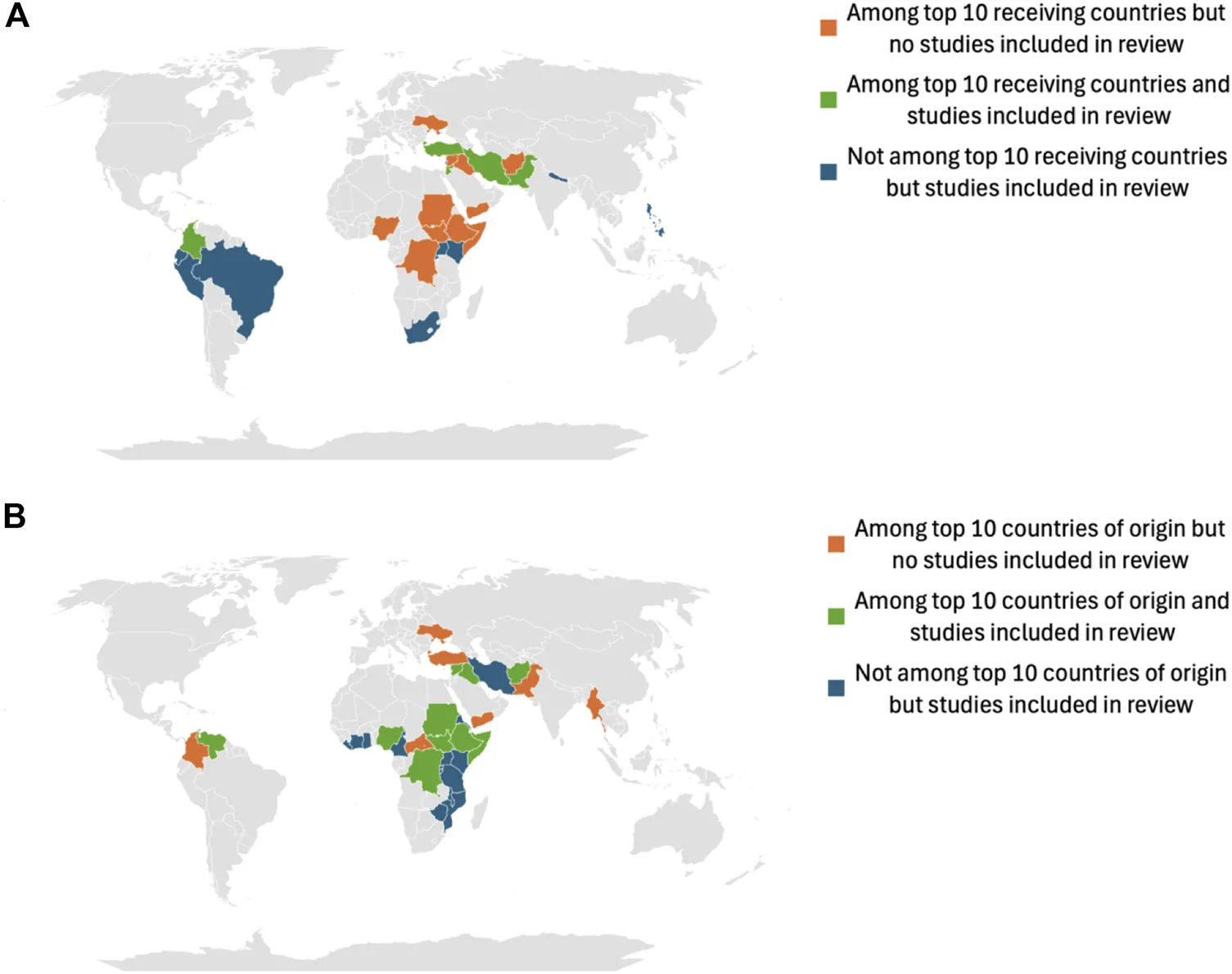
Geographical distribution of receiving countries (panel **(A)**) and refugees’ countries of origin (panel **(B)**), illustrating (i) missing literature on countries most affected in orange, (ii) studied countries among the most affected in green and (iii) studied countries not among the top 10 most affected in blue (Basel, Switzerland, 2025).

No literature on internally displaced people was identified to be included in this review. This is especially striking, given that nearly 60% of all forcibly displaced people remain within their own country [1]. It is, however, possible that some research on discrimination against internally displaced people has been conducted but was not included in this review due to the lack of differentiation of the migrant population or the rather narrow scope of the review (see Section *Strengths and Limitations*).

### Perceived discrimination in health settings

Most studies in the review applied an explorative qualitative approach, primarily investigating barriers to healthcare access without specifically focusing on discrimination. As a result, generalizable data on prevalence and context of discrimination in healthcare in LMICs among forcibly displaced is lacking. Although five of the studies used quantitative methods, none used standardized tools such as the Discrimination in Medical Settings Scale [61] to systematically assess perceived discrimination. The results of the studies in the review are therefore very heterogeneous, ranging from mere mentioning of perceived discrimination to rich descriptions of displaced people experiencing discriminating attitudes, mistreatment, verbal abuse or disrespectful behavior by healthcare providers when seeking care in the host country [20, 24, 26–28, 31, 33–35, 37–45]. Several studies reported perceived discrimination in healthcare as a barrier to accessing care. Refugees who were afraid of discrimination or were dissatisfied with the service avoided certain facilities or delayed seeking care until their health situation had reached a critical stage [20, 27, 34, 39, 40, 42, 50, 51]. Stigma and mistreatment of refugees by healthcare workers also lead to mistrust and confidentiality concerns [36, 52, 53]. Some refugees expressed feeling worthless due to exclusion, others reported increased trauma and stress or a compromise of emotional wellbeing [33, 35, 46].

The quantitative results show that 10%–44% of the refugees reported experiencing discrimination while seeking healthcare [20, 30, 31, 37]. While these numbers are comparable to findings from recent studies on discrimination in medical settings across different populations in the United States, where 21%–40% of respondents reported experiencing discrimination in healthcare [58, 59, 62], a study published in 2017 on perceived discrimination in primary healthcare across various European countries (and populations) reported slightly lower rates, with 7% (1.4%–12.8%) of the respondents indicating they had felt discriminated against [54].

Overall, the existing evidence suggests that perceived discrimination in medical settings poses a significant barrier to refugees receiving high-quality healthcare, however, the true prevalence, determinants and impacts remain insufficiently understood due to methodological limitations and the lack of standardized measurement approaches.

### Underlying reasons for discrimination

In the identified literature, the reasons for perceived discrimination often remain unclear. In many cases, it was not possible to distinguish whether the perceived unfair treatment was based on actual discrimination or on the perception of some other form of mistreatment unrelated to personal traits and affecting all patient populations (e.g., general bad attitude of certain health staff towards all patients, lack of commodities for all patients). Some studies in our review included perspectives from healthcare providers or key informants from refugee organizations, offering an additional viewpoint and, to some extent, possible explanations for the perceived unfair treatment of refugees by healthcare providers. These included, among others, feelings of resentment toward refugees, work overload, a general scarcity of resources, discriminatory policies and language barriers [25, 29, 37, 38, 40–42, 45, 51]. However, the study designs often did not allow direct comparison or objectification of the answers (e.g., no comparison group was used, different questions were asked, different methodologies were applied or a qualitative approach resulted in heterogeneous narratives). Although the effect on the refugee’s health outcomes and health-seeking behavior is likely to be the same, regardless of the underlying cause of the perceived discrimination or whether the discriminatory acts can be objectively verified [50, 63], understanding the underlying reasons for perceived discrimination is essential to develop effective strategies to tackle discriminatory behavior and improve healthcare for displaced people. This underscores the need for methodologically robust, comparative and mixed-method research to identify and address the underlying reasons for perceived discrimination in healthcare settings.

### Reducing discrimination in healthcare

Effectively addressing discrimination in healthcare will require a coordinated, evidence-informed and context-sensitive approach that recognizes the complexity of migration, discrimination and health systems [64]. Based on the studies in our review, several general recommendations can be drawn to reduce discrimination in healthcare. Firstly, enhancing intercultural competencies among healthcare providers through training on migrants’ legal rights, cultural diversity and anti-discrimination, as well as integrating migrants into the healthcare workforce, is needed [20, 24, 27, 28, 33, 35, 41, 43–47, 49, 50, 65]. Secondly, strengthening health systems is essential, including ensuring adequate staffing, supplies and infrastructure, as well as employing professional interpreters to support the delivery of high-quality care for all [20, 26, 28, 29, 32, 36, 38, 40]. Thirdly, to protect migrants from violence and deportation and to reduce xenophobia in healthcare, inclusive policies must be developed and promoted and international guidelines and initiatives should be adapted to national contexts [24, 26, 27, 30, 33, 38, 39, 41–44, 47, 48, 50–53]. Finally, further research is needed to understand healthcare providers’ justifications for behaviors perceived as discriminatory, to explore specific barriers to healthcare access faced by migrant populations and to examine the underlying causes of marginalization and structural violence [24, 25, 32, 34, 37].

However, as pointed out by several authors in the *Lancet* Series on Racism, Xenophobia, Discrimination and Health, it should not be forgotten that healthcare is just one of the institutions in which discrimination has an impact on health outcomes of migrants and other minorities [4, 65, 66]. Therefore, it will not be sufficient to merely try to “cure” the causes of discrimination in healthcare by improving access to services and enhancing cultural sensitivity among healthcare providers alone. Ultimately, it will be necessary to address discrimination as a fundamental social determinant of health and tackle the underlying systems of categorization, marginalization and oppression, the overall root causes of discrimination, racism and xenophobia [4, 65, 66].

### Recommendations for future research

Drawing on this literature review, the following recommendations aim to strengthen future research on discrimination against forcibly displaced populations in healthcare. Studies should use validated measures of self-reported discrimination, clearly define study populations and migrant subgroups, and include appropriate control groups to enable comparative analysis. Incorporating multiple perspectives, including those of healthcare workers and third-party informants such as representatives of refugee organizations, can improve contextual understanding. Research should assess the prevalence, settings, forms, and drivers of discrimination by examining where, when, why, and against whom it occurs, as well as who perpetrates it. Related data analyses should control for key confounders, including demographic, legal, socioeconomic and health-related factors. These findings should be interpreted within relevant historical, political and policy contexts. Publishing results, particularly from multi-setting or cross-country studies, will strengthen the global comparative evidence base.

### Strengths and limitations

The scoping review followed the frameworks of Arksey and O’Malley, Levac et al., and the PRISMA-ScR guidelines to ensure a systematic and transparent process [17–19]. PubMed and Web of Science were used, with MeSH terms applied in PubMed to expand the search; the absence of these terms in Web of Science may have limited the number of retrieved studies. Inclusion and exclusion criteria were defined, refined after screening the first 100 studies, and consistently applied. Two authors independently screened titles and abstracts, resolving disagreements through discussion to minimize selection bias. Only English, peer-reviewed primary studies published between March 2010 and March 2025 were included. This may have excluded earlier research, grey literature, non-English studies, and English reviews covering non-English work. The language restriction may have introduced a geographical and cultural bias into the findings. However, we expect only a small percentage of the literature to be unavailable in English. Studies had to clearly define migrant populations and provide results specific to forcibly displaced people, which may have excluded research where refugees or internally displaced persons were not distinguished from voluntary migrants. Focusing solely on discrimination or barriers to healthcare access may have omitted broader studies of migrant experiences. As is standard in scoping reviews, no formal quality assessment was conducted; however, limiting inclusion to peer-reviewed studies ensured a baseline level of methodological quality.

### Conclusion

Overall, this review highlights the scarcity of robust, comparable and context-specific data on healthcare discrimination as perceived by displaced populations in LMICs. Many of the most affected host countries are underrepresented, as are their populations. Nevertheless, it provides valuable insights into the existing evidence, suggesting that perceived discrimination in medical settings poses a significant barrier to refugees receiving high-quality healthcare. This evidence can be used to generate hypotheses for future studies and allows authors to take on a mixed-method approach to examine the prevalence, circumstances and impacts of discrimination on health outcomes and health seeking behavior. To improve access to and the quality of care for migrants, future research findings should be incorporated in national policies, guidelines and intervention programs that promote inclusion and equality in healthcare. Meanwhile, efforts should focus on strengthening health systems, enhancing intercultural competencies and advancing inclusive policies that ensure respectful, high-quality care for forcibly displaced populations and other migrants.
