# Supplementary material for: Discrimination in medical settings as perceived by forcibly displaced people within low- and middle-income countries: a scoping literature review
Source: Public Health Rev. 2026 Oct 5;47:1609595. doi: 10.3389/phrs.2026.1609595 (PMC13677560; doi:10.3389/phrs.2026.1609595)
Supplement: Supplementary file 1 [file DataSheet1.PDF]

Supplementary File 1 – Scoping Literature Review Protocol and Methods

Discrimination in Medical Settings as perceived by Forcibly Displaced People within Low- and Middle-Income Countries: Scoping Literature Review Protocol and Methods

Abstract

Objectives: Forcibly displaced people are among the most vulnerable populations seeking care. Understanding barriers such as perceived discrimination is critical to ensuring access to high-quality care. This review aims to provide an overview of scientific literature on discrimination faced by forcibly displaced people in healthcare settings in low- and middle-income countries (LMICs).
Methods: We searched PubMed and Web of Science for peer-reviewed original studies published between March 2010 and March 2025. Screening, data extraction and reporting followed the PRISMA guidelines for scoping reviews.
Results: Thirty-one studies met the inclusion criteria. Most (n=26) applied qualitative methods. Quantitative findings showed that 10-44% of refugees reported discrimination in healthcare. Qualitative studies described abuse, disparities in care and costs, and negative impacts on mental health and health-seeking behavior related to perceived discrimination.
Conclusion: The review revealed significant gaps in research on discrimination against forcibly displaced people in LMICs, emphasizing the urgent need for more robust evidence to inform policymakers. Existing research demonstrates the importance of strengthening health systems, intercultural competence and inclusive policies to ensure respectful, high-quality care for forcibly displaced populations and other migrants.

Keywords: Health care, low- and middle-income countries, migrant, perceived discrimination, refugee

1. Introduction

The UN Refugee Agency (UNHCR) estimates that at the end of 2024 the number of people forced to flee persecution, conflict, violence, human rights violations and events seriously disturbing public order exceeded 120 million [1]. This figure includes both, internally displaced people, meaning people who remained within their own country, and people who have crossed borders and fled to another country (refugees and asylum-seekers). With over 73 million people affected, internally displaced people account for the majority of the forcibly displaced [2]. Meanwhile, the refugee population has more than doubled over the past decade and risen above 42 million [3]. Additionally, more than 8 million people were waiting for a decision on their asylum-request [4]. Low- and middle-income countries (LMICs) host the majority of all forcibly displaced people, including 75% of (international) refugees [1].

According to the International Organization for Migration (IOM) the common lay understanding of a "migrant" is someone "who moves away from their place of usual residence, whether within a country or across an international border, temporarily or permanently, and for a variety of reasons". However, there is no internationally accepted definition for the term "migrant" [5]. This term is therefore very broad and does not discriminate between people who move voluntarily, for example, for work-related reasons or people who have been forced to leave their homes due to conflict, persecution, human rights abuses or natural disasters.

The term "forcibly displaced people" is clearer, meaning internally displaced, asylum-seekers and refugees [1]. Internally displaced persons are people who have been forced or obliged to flee their homes or places of habitual residence due to armed conflicts, generalized violence, human rights abuses and natural or human-made disasters. However, unlike asylum-seekers and refugees, they have not crossed an international border and remain in their country of origin [2]. An "asylum-seeker" is a person who has fled their country and is seeking international protection [4]. As soon as asylum has been granted the asylum-seeker receives the legal status of a refugee [3]. However, according to the IOM it does not depend on the decision of the host country if someone is a refugee or not. Instead, there's a set of criteria that must be met (1951 Convention). Anyone who fulfils the criteria is a refugee and should therefore get recognition of the host country [5].

According to the American Psychological Association discrimination is "the unfair treatment or prejudicial treatment of people and groups based on characteristics such as race, gender, age

or sexual orientation" [6]. There are various ways in which patients may experience discrimination in the health care sector. Including, but not limited to, rude comments, neglect, poorer quality of treatment or limited access to health care facilities [7, 8, 9]. Several studies have shown that discrimination in medical settings can act as a barrier to health care access. For example, the expectation of being discriminated against may discourage people from seeking health care in the first place [10-13]. Furthermore, being discriminated against can be a reason for early self-discharge from the hospital or lower medication adherence [14, 15]. Since forcibly displaced people belong to the most vulnerable among all patients seeking health care [16], understanding and evaluating potential barriers to health care in their resettlement location, including perceived discrimination, is essential to ensure access to high-quality care for all.

Up to date, to our knowledge, there are no scoping reviews focusing on discrimination in health care of forcibly displaced people in LMICs. Therefore, it remains unclear to what extent discrimination in medical settings as perceived by refugees and internally displaced people in LMICs has been investigated. Having this knowledge is particularly important since according to the UN Refugee Agency (UNHCR) not only the majority of all forcibly displaced people, which includes internally displaced people, but also 75% of (international) refugees live in LMICs [1].

2. Objectives and Research Questions

The objective of this scoping review is to map out the state of peer-reviewed English literature on discrimination in the medical setting as perceived by forcibly displaced people in LMICs.

Therefore, the research questions of the scoping review are:

- To what extent has perceived discrimination experienced by refugees and internally displaced people been investigated in medical settings in LMICs?
- What are the remaining knowledge gaps?
- What research methods have been used?
- What were the main research questions?
- What were the key findings regarding perceived discrimination against forcibly displaced people in healthcare settings in LMICs?
- What are the key recommendations presented in the papers?

3. Methods

Since the main aim is to map the state of existing literature and identify gaps in the current literature, the review will be conducted as a "Scoping Study" [17]. The methodology of the review will follow the approach of Arksey & O'Malley and the recommendations on the framework of Levac et. al [17, 18]. Therefore, the review will be carried out according to the following stages: (i) identification of relevant studies; (ii) selection of studies; (iii) extraction of data; and (iv) summary and report of results. Reporting will be conducted according to the "Preferred Reporting Items for Systematic reviews and Meta-Analyses extension for Scoping Reviews (PRISMA-ScR): Checklist and Explanation" [19].

3.1. Search Terms and Strategy

Relevant peer-reviewed studies published between March 11th 2010 and March 10th 2025 will be identified using PubMed and Web of Science. The search string consists of four blocks around the following terms: (i) Refugee; (ii) Healthcare; (iii) Discrimination; and (iv) LMICs. The search string was adapted to the specific features of the two databases (Table 1). All hits with an English abstract will be included, irrespective of the language of the full text. LMICs were defined according to the World Bank ("World Bank Country and Lending Groups", March 2025 [20]).

Table 1: Search term blocks with detailed search terminology (developed for PubMed).

**Refugee**	refugee* OR migrant* OR migration OR asylum seeker* OR "refugees" [MeSH Terms] OR "transients and migrants" [MeSH Terms]
**Healthcare**	healthcare OR "health care" OR health care quality OR "medical setting" OR "dental care" OR "mental care" OR hospital* OR emergency servic* OR "medical staff" OR "health staff" OR "health care worker" OR "health system" OR "health seeking" OR "delivery of health care" [MeSH Terms] OR "mental health services" [MeSH Terms] OR "health facilities" [MeSH Terms] OR "emergency medical services" [MeSH Terms] OR "quality of health care" [MeSH Terms]
**Discrimination**	discrimination OR racism OR inequalit* OR disparit* OR xenophobia OR "social discrimination" [MeSH Terms] OR "racism" [MeSH Terms]
**LMICs**	developing countr* OR low middle income countr* OR resource-limited setting* OR "developing countries" [MeSH Terms] OR name of countries as listed by World Bank Country and Lending Groups [20] (see Search String for PubMed and Search String for Web of Science below)

Search String for PubMed: (refugee* [Title/Abstract] OR migrant* [Title/Abstract] OR migration [Title/Abstract] OR asylum seeker* [Title/Abstract] OR "refugees" [MeSH Terms] OR "transients and migrants" [MeSH Terms]) **AND** (healthcare [Title/Abstract] OR "health

care” [Title/Abstract] OR health care quality [Title/Abstract] OR “medical setting” [Title/Abstract] OR “dental care” [Title/Abstract] OR “mental care” [Title/Abstract] OR hospital* [Title/Abstract] OR emergency servic* [Title/Abstract] OR “medical staff” [Title/Abstract] OR “health staff” [Title/Abstract] OR “health care worker” [Title/Abstract] OR “health system” [Title/Abstract] OR “health seeking” [Title/Abstract] OR “delivery of health care” [MeSH Terms] OR “mental health services” [MeSH Terms] OR “health facilities” [MeSH Terms] OR “emergency medical services” [MeSH Terms] OR “quality of health care” [MeSH Terms]) **AND** (discrimination OR racism OR inequalit* OR disparit* OR xenophobia OR “social discrimination” [MeSH Terms] OR “racism” [MeSH Terms]) **AND** (developing countr* OR low middle income countr* OR resource-limited setting* OR “developing countries” [MeSH Terms] **OR** Afghanistan OR Burkina Faso OR Burundi OR Central African Republic OR Congo OR Eritrea OR Ethiopia OR Gambia OR Guinea-Bissau OR Korea OR Liberia OR Madagascar OR Malawi OR Mali OR Mozambique OR Niger OR Rwanda OR Sierra Leone OR Somalia OR South Sudan OR Sudan OR Syria Arab Republic OR Togo OR Uganda OR Yemen OR Angola OR Bangladesh OR Benin OR Bhutan OR Bolivia OR Cabo Verde OR Cambodia OR Cameroon OR Comoros OR Congo OR Côte d'Ivoire OR Djibouti OR Egypt OR Eswatini OR Ghana OR Guinea OR Haiti OR Honduras OR India OR Jordan OR Kenya OR Kiribati OR Kyrgyz Republic OR Lao OR Laos OR Lebanon OR Lesotho OR Mauritania OR Micronesia OR Morocco OR Myanmar OR Nepal OR Nicaragua OR Nigeria OR Pakistan OR Papua New Guinea OR Philippines OR Samoa OR São Tomé and Principe OR Senegal OR Solomon Islands OR Sri Lanka OR Tajikistan OR Tanzania OR Timor-Leste OR Tunisia OR Uzbekistan OR Vanuatu OR Vietnam OR West Bank OR Gaza OR Zambia OR Zimbabwe OR Albania OR Algeria OR Argentina OR Armenia OR Azerbaijan OR Belarus OR Belize OR Bosnia and Herzegovina OR Botswana OR Brazil OR China OR Colombia OR Costa Rica OR Cuba OR Dominica OR Dominican Republic OR Ecuador OR El Salvador OR Equatorial Guinea OR Fiji OR Gabon OR Georgia OR Grenada OR Guatemala OR Indonesia OR Iran OR Iraq OR Jamaica OR Kazakhstan OR Kosovo OR Libya OR Malaysia OR Maldives OR Marshall Islands OR Mauritius OR Mexico OR Moldova OR Mongolia OR Montenegro OR Namibia OR North Macedonia OR Paraguay OR Peru OR Serbia OR South Africa OR St. Lucia OR St. Vincent and the Grenadines OR Suriname OR Thailand OR Tonga OR Türkiye OR turkey OR Turkmenistan OR Tuvalu OR Ukraine)

→ 1741 search hits in PubMed 2010/03/11-2025/03/10

Search String for Web of Science: (refugee* OR migrant* OR migration OR asylum seeker* OR “transients and migrants”) **AND** (healthcare OR “health care” OR health care quality OR “medical setting” OR “dental care” OR “mental care” OR hospital* OR emergency servic* OR “medical staff” OR “health staff” OR “health care worker” OR “health system” OR “health seeking” OR “delivery of health care” OR “mental health services” OR “health facilities” OR “emergency medical services” OR “quality of health care”) **AND** (discrimination OR racism OR inequalit* OR disparit* OR xenophobia OR “social discrimination”) **AND** (developing countr* OR low middle income countr* OR resource-limited setting* **OR** Afghanistan OR Burkina Faso OR Burundi OR Central African Republic OR Congo OR Eritrea OR Ethiopia OR Gambia OR Guinea-Bissau OR Korea OR Liberia OR Madagascar OR Malawi OR Mali OR Mozambique OR Niger OR Rwanda OR Sierra Leone OR Somalia OR South Sudan OR Sudan OR Syria Arab Republic OR Togo OR Uganda OR Yemen OR Angola OR Bangladesh OR Benin OR Bhutan OR Bolivia OR Cabo Verde OR Cambodia OR Cameroon OR Comoros OR Congo OR Côte d'Ivoire OR Djibouti OR Egypt OR Eswatini OR Ghana OR Guinea OR Haiti OR Honduras OR India OR Jordan OR Kenya OR Kiribati OR Kyrgyz Republic OR Lao OR Laos OR Lebanon OR Lesotho OR Mauritania OR Micronesia OR Morocco OR Myanmar OR Nepal OR Nicaragua OR Nigeria OR Pakistan OR Papua New Guinea OR Philippines OR Samoa OR São Tomé and Principe OR Senegal OR Solomon Islands OR Sri Lanka OR Tajikistan OR Tanzania OR Timor-Leste OR Tunisia OR Uzbekistan OR Vanuatu OR Vietnam OR West Bank OR Gaza OR Zambia OR Zimbabwe OR Albania OR Algeria OR Argentina OR Armenia OR Azerbaijan OR Belarus OR Belize OR Bosnia and Herzegovina OR Botswana OR Brazil OR China OR Colombia OR Costa Rica OR Cuba OR Dominica OR Dominican Republic OR Ecuador OR El Salvador OR Equatorial Guinea OR Fiji OR Gabon OR Georgia OR Grenada OR Guatemala OR Indonesia OR Iran OR Iraq OR Jamaica OR Kazakhstan OR Kosovo OR Libya OR Malaysia OR Maldives OR Marshall Islands OR Mauritius OR Mexico OR Moldova OR Mongolia OR Montenegro OR Namibia OR North Macedonia OR Paraguay OR Peru OR Serbia OR South Africa OR St. Lucia OR St. Vincent and the Grenadines OR Suriname OR Thailand OR Tonga OR Türkiye OR turkey OR Turkmenistan OR Tuvalu OR Ukraine)

→ 722 search hits in Web of Science 2010/03/11-2025/03/10 (when putting search terms for refugee, health care and specific country names in title & abstract and terms for discrimination and LMICs in all fields)

3.2. Peer-Reviewed Literature Screening

Parallel screening of all titles and abstracts will be conducted by P.K. and K.G. using EndNote (version 21.5, Clarivate Analytics, United States) for data management and Covidence (2025 Covidence, Veritas Health Innovation, Australia) to identify relevant studies. Discrepancies will be resolved in discussion and disagreements or uncertainties will be discussed with M.S.W. as senior researcher. Duplicates will automatically be detected and removed by Covidence, in addition, if needed, duplicates will be manually removed. Full paper screening will be carried out by K.G. Final in- and exclusion of papers will be discussed with P.K. Studies will be considered eligible if (i) they included data on discrimination in medical settings as perceived by forcibly displaced people in LMICs in the result section (ii) the aim of the study was to investigate discrimination in health care or to explore barriers to health care and (iii) full text was available in English within the rights of the University of Basel. As study population all forcibly displaced people of all ages will be considered. Studies will be excluded if the studied migrant populations are not clearly defined as forcibly displaced, if results on forcibly displaced are not available independently from other migrant or non-migrant populations or if discrimination is reported by anyone outside of the migrant population (e.g. health care worker's perspectives). All research methodologies will be included, provided the studies have been peer-reviewed and published primary data. No restrictions regarding the origin of the authors or the time frame of the investigations are set. Table 2 provides an overview of the key characteristics of the scoping review.

Table 2: Overview of characteristics of scoping literature review including source of information, limiting parameters, study population, in- and exclusion criteria, tools used to conduct the review and key words.

**Characteristics of literature review**	
**Type of review**	Scoping Literature Review
**Information sources**	PubMed and Web of Science
**Time frame publication date**	March 11th 2010 – March 10th 2025
**Time frame studied population**	No limitations
**Author's location**	No limitations
**Host countries**	All LMICs, according to the World Bank Country and Lending Groups (March 2025)
**Study population**	All forcibly displaced people (refugees, asylum seekers, internally displaced people), all ages
**Types of literature**	• Peer reviewed papers presenting primary data, all types of methodologies<br>• Reviews were excluded but used to identify papers presenting primary data that were not initially identified through literature search
**Inclusion criteria**	• Data on discrimination in medical settings as perceived by forcibly displaced people, all types of health care (primary to tertiary, dental, mental …)<br>AND<br>• Aim of study to investigate discrimination in health care AND/OR exploring barriers to health care access / inequalities in health care<br>AND<br>• Full text retrievable in English with the access rights of the University of Basel
**Exclusion criteria**	• Described discrimination not from the perspective of the migrant<br>• Results not primary data<br>• Literature not peer-reviewed<br>• Grey literature (e.g. comments, articles, policies)<br>• Unclear or non-migrant study population, voluntary migration (e.g. labor migration)<br>• Data exclusively focusing on HICs or not possible to extract results for LMICs independently<br>• Full text paper not retrievable in English within the access rights of the University of Basel
**Tools**	• End Note (version 21.5, Clarivate Analytics, United States)<br>• Covidence (2025 Covidence, Veritas Health Innovation, Australia)<br>• Microsoft Excel (version 16.98, Microsoft Corporation, United States)
**Key words**	Health care, low- and middle-income countries, migrant, perceived discrimination, refugee

3.3. Data Analysis

Data from the papers remaining of the full-text analysis will be extracted by K.G. A data charting form was developed for this purpose by K.G., with inputs from M.S.W., using a Microsoft Excel (version 16.98, Microsoft Corporation, United States) spread sheet. The following variables were included in the charting form: title, first author, host countries studied, journal of publication, year of publication, study population, number of participants, age of population, type of study and methods (used for analysis of discrimination), aim of the study, main findings, conclusion and main recommendations. For data analysis and generating of tables and charts Microsoft Excel will be used. A descriptive approached will be applied to characterize included studies according to their year of publication, geographical scope, study population, methodological approach and main objectives. Key findings and conclusions / recommendations of the studies will be grouped into sub-themes using an inductive coding approach.

4. References

1. United Nations High Commissioner for Refugees. Global Trends. 2024 [last accessed 21.01.2026]. Available from: https://www.unhcr.org/global-trends
2. United Nations High Commissioner for Refugees. What is internal displacement? 2024 [last accessed 21.01.2026]. Available from: https://www.unhcr.org/about-unhcr/who-we-protect/internally-displaced-people
3. United Nations High Commissioner for Refugees. Who is a “refugee”? 2024 [last accessed 21.1.2026]. Available from: https://www.unhcr.org/about-unhcr/who-we-protect/refugees
4. United Nations High Commissioner for Refugees. Who is an “asylum-seeker”? 2024 [last accessed 21.01.2026]. Available from: https://www.unhcr.org/about-unhcr/who-we-protect/asylum-seekers
5. International Organization for Migration. Glossary on migration. 2019 [last accessed 21.01.2026]. Available from: https://publications.iom.int/system/files/pdf/iml_34_glossary.pdf
6. Association A.P. Discrimination: What it is and how to cope. 2019 [last accessed 21.01.2026]. Available from: https://www.apa.org/topics/racism-bias-discrimination/types-stress
7. Mangrio E., Sjogren Forss K. Refugees' experiences of healthcare in the host country: a scoping review. *BMC Health Services Research*. 2017;17(1):814. doi: 10.1186/s12913-017-2731-0.
8. Pattillo M., Stieglitz S., Angoumis K., Gottlieb N. Racism against racialized migrants in healthcare in Europe: a scoping review. *International Journal for Equity in Health*. 2023;22(1):201. doi: 10.1186/s12939-023-02014-1.
9. Abubakar I., Aldridge R.W., Devakumar D., Orcutt M., Burns R., Barreto M.L., et al. The UCL-Lancet Commission on Migration and Health: the health of a world on the move. *Lancet*. 2018;392(10164):2606-54. doi: 10.1016/S0140-6736(18)32114-7.

10. Gonzales K.L., Harding A.K., Lambert W.E., Fu R., Henderson W.G. Perceived experiences of discrimination in health care: a barrier for cancer screening among American Indian women with type 2 diabetes. *Womens Health Issues*. 2013;23(1):e61-7. doi: 10.1016/j.whi.2012.10.004.
11. Bawadi H., Al-Hamdan Z., Ahmad M.M. Needs of Migrant Arab Muslim Childbearing Women in the United Kingdom. *Journal of Transcultural Nursing*. 2020;31(6):591-7. doi: 10.1177/1043659620921219.
12. McLean C., Campbell C., Cornish F. African-Caribbean interactions with mental health services in the UK: experiences and expectations of exclusion as (re)productive of health inequalities. *Social Science & Medicine*. 2003;56(3):657-69. doi: 10.1016/s0277-9536(02)00063-1.
13. Worth A., Irshad T., Bhopal R., Brown D., Lawton J., Grant E., et al. Vulnerability and access to care for South Asian Sikh and Muslim patients with life limiting illness in Scotland: prospective longitudinal qualitative study. *BMJ*. 2009;338:b183. doi: 10.1136/bmj.b183.
14. Askew D.A., Foley W., Kirk C., Williamson D. "I'm outta here!": a qualitative investigation into why Aboriginal and non-Aboriginal people self-discharge from hospital. *BMC Health Services Research*. 2021;21(1):907. doi: 10.1186/s12913-021-06880-9.
15. Cuffee Y.L., Hargraves J.L., Rosal M., Briesacher B.A., Schoenthaler A., Person S., et al. Reported racial discrimination, trust in physicians, and medication adherence among inner-city African Americans with hypertension. *American Journal of Public Health*. 2013;103(11):e55-62. doi: 10.2105/AJPH.2013.301554.
16. World Health Organization. Refugee and Migrant health. 2022 [last accessed 21.01.2026]. Available from: https://www.who.int/news-room/fact-sheets/detail/refugee-and-migrant-health?utm_source=chatgpt.com
17. Arksey H., O'Malley L. Scoping studies: towards a methodological framework. *International Journal of Social Research Methodology*. 2005;8(1):19-32. doi: 10.1080/1364557032000119616.
18. Levac D., Colquhoun H., O'Brien K.K. Scoping studies: advancing the methodology. *Implementation Science*. 2010;5:69. doi: 10.1186/1748-5908-5-69.
19. Tricco A.C., Lillie E., Zarin W., O'Brien K.K., Colquhoun H., Levac D., et al. PRISMA Extension for Scoping Reviews (PRISMA-ScR): Checklist and Explanation. *Annals of Internal Medicine*. 2018;169(7):467-73. doi: 10.7326/M18-0850.
20. The World Bank. World Bank Country and Lending Groups. 2025 [last accessed 26.01.2026]. Available from: https://datahelpdesk.worldbank.org/knowledgebase/articles/906519-world-bank-country-and-lending-groups
