# Supplementary material for: Discrimination in medical settings as perceived by forcibly displaced people within low- and middle-income countries: a scoping literature review
Source: Public Health Rev. 2026 Oct 5;47:1609595. doi: 10.3389/phrs.2026.1609595 (PMC13677560; doi:10.3389/phrs.2026.1609595)
Supplement: Supplementary file 2 [file DataSheet2.PDF]

Supplementary File 2 – Table of Included Studies (Objectives and Methods)

Table: Overview of studies included in review with methodology and main objectives (pink = woman only, blue = discrimination in health care as main objective)

First author, Year, Title	Host country	Study population	Methods	Objectives short	Objectives
Arnold, C, 2014, A qualitative exploration of access to urban migrant healthcare in Nairobi, Kenya	Kenya	Migrants from Côte d'Ivoire, DRC, Rwanda, Tanzania, Uganda, (Refugees, asylum-seekers and irregular migrants)	Qualitative, FGD	Barriers to health care access	The purpose of this project was to explore barriers to accessing healthcare experienced by urban migrants and Kenyans living in areas considered to be spaces of vulnerability in Nairobi.
Ay, M, 2016, The Perceived Barriers of Access to Health Care Among a Group of Non-camp Syrian Refugees in Jordan	Jordan	Syrian refugees	Quantitative, questionnaire	Barriers to health care access	The aims of this study were to identify the perceived health care service needs, accessibility of services, and barriers to access among a group of Syrian refugees living outside the camps in four governorates of Jordan and to compare the differences in access among them.
Benavente, P, 2025, Deployment and uptake of COVID-19 vaccines for refugees and migrants in regular and irregular situations: a mixed-method multicountry study	Ecuador, Nepal, Pakistan, Philippines and Rwanda	Refugees and other migrants	Quantitative, questionnaire	Barriers to health care access	This study aimed to identify barriers and facilitators and estimate vaccination coverage among refugees and migrants in low- and middle-income countries, emphasizing inclusive policies for effective rollout.
Bridi, L, 2023, Attitudes toward dementia and cognitive aging among Syrian refugees resettled in Jordan: a qualitative study	Jordan	Syrian refugees	Qualitative, FGD	Barriers to health care access	This study aims to document aging Syrian refugees' knowledge and attitudes toward dementia and cognitive aging, along with their experiences with the Jordanian healthcare system.
Chirau, T. J, 2024, Access to healthcare by undocumented Zimbabwean migrants in post-apartheid South Africa	South Africa	Undocumented Zimbabwean migrants	Qualitative, interviews	Barriers to health care access	This article aimed to describe and understand the experiences, challenges and health-seeking alternatives of undocumented Zimbabwean migrants in accessing healthcare services in Nellmapius in Pretoria.
Crush, J, 2014, Medical Xenophobia and Zimbabwean Migrant Access to Public Health Services in South Africa	South Africa	Zimbabwean migrants including asylum seekers	Qualitative, interviews and FGD	Discrimination in healthcare	This paper examines the experience of medical xenophobia by recent (post-2005) Zimbabwean migrants who are living and working in South Africa.
Dadras, O, 2020, "It is good, but I can't afford it …" potential barriers to adequate prenatal care among Afghan women in Iran: a qualitative study in South Tehran	Iran	Afghan refugee woman	Qualitative, interviews	Barriers to health care access	This paper explores the potential barriers to prenatal care among Afghan women in Iran

First author, Year, Title	Host country	Study population	Methods	Objectives short	Objectives
Dahlberg, M, 2023, Conceptualizing xenophobia as structural violence in the lives of refugee women in Gauteng, South Africa	South Africa	African refugee/asylum seeking women (Burundi, DRC, Eritrea, Ethiopia, Somalia and Zimbabwe)	Qualitative, interviews and FGD	Barriers to health care access and discrimination in health care	Drawing on in-depth qualitative interview data with refugee women in Gauteng, South Africa, we explore the empirical utility of the concept of structural violence in shaping refugee women's everyday experiences of xenophobia through three analytical themes: (a) unequal access to resources (b) constrained agency and (c) dehumanization. We explore these mechanisms through their interactions and intersections with three public institutions and their role in generating and nurturing xenophobia: The Department of Home Affairs, The South African Police Service and Public Hospitals.
Doğan, N, 2019, Syrian refugees' experiences with mental health services in Turkey: "I felt lonely because I wasn't able to speak to anyone"	Turkey	Syrian refugees	Qualitative, FGD	Barriers to health care access	This study examined Syrian refugee adults' experiences with mental health services due to a mental complaint.
Genç, H. D, 2024, Migrants' access to healthcare services: evidence from fieldwork in Turkey	Turkey	Refugees and asylum seekers from Afghanistan, Iran, Syria	Qualitative, interviews	Barriers to health care access	The study is built on data collected through in-depth interviews with migrants residing in Turkey. In order to capture their experiences and explore the factors influencing access to healthcare services, it focuses on six groups: regular and irregular migrants; spousal migrants; international retirement migrants; international students; and refugees and asylum seekers.
Kabakian-Khasholian, T, 2022, "A person who does not have money does not enter": a qualitative study on refugee women's experiences of respectful maternity care	Lebanon	Palestinian, Iraqi and Syrian refugee woman	Qualitative, interviews	Discrimination in healthcare	This study aims at presenting Palestinian, Iraqi and Syrian refugee women's experiences, understanding and interpretation of disrespect and mistreatment during childbirth in hospitals in Lebanon.
Karaki, F. M, 2021, Noncommunicable Disease and Health Care-Seeking Behavior Among Urban Camp-Dwelling Syrian Refugees in Lebanon: A Preliminary Investigation	Lebanon	Syrian refugees	Qualitative, interviews	Barriers to health care access	We sought to assess NCD burden and explore barriers to health care access among urban-dwelling SRs living in BBRC, as perceived by both SRs and health care workers (HCWs). We also sought to elucidate the attitudes of SRs and HCWs toward the quality of their health and health care services.
Khalifeh, R, 2023, Experiences of Cultural Differences, Discrimination, and Healthcare Access of Displaced Syrians (DS) in Lebanon: A Qualitative Study	Lebanon	Syrian refugees	Qualitative, interviews	Barriers to health care access and discrimination in health care	The study aims to examine cultural differences and discrimination as difficulties encountered by displaced Syrians when using the Lebanese healthcare system, and to evaluate the equity of displaced Syrians access to health services in Lebanon.

First author, Year, Title	Host country	Study population	Methods	Objectives short	Objectives
Khalifeh, R, 2023, Healthcare bias and health inequalities towards displaced Syrians in Lebanon: a qualitative study	Lebanon	Syrian refugees	Qualitative, FGD	Barriers to health care access and discrimination in health care	This study aims to identify displaced Syrians perception of healthcare biases in the Lebanese healthcare system, and its consequences on displaced Syrians accessing and receiving quality healthcare in Lebanon.
Kibiribiri, E, 2016, Exploring disparities in prenatal care between refugees and local South African women	South Africa	African refugee women (Burundi, Cameroon, DRC, Ghana, Kenya, Liberia, Malawi, Mozambique, Rwanda, Somalia, Tanzania, Uganda, Zimbabwe)	Quantitative, questionnaire	Discrimination in healthcare	To explore possible disparities in prenatal care between refugees and South African women attending public health facilities in an urban setting in South Africa.
Logie, C. H, 2021, Intersecting stigma and HIV testing practices among urban refugee adolescents and youth in Kampala, Uganda: qualitative findings	Uganda	Refugee adolescents from Burundi, DRC, Rwanda, Sudan	Qualitative, FGD	Barriers to health care access and discrimination in health care	There are knowledge gaps regarding HIV testing facilitators and barriers, including HIV and intersecting stigmas, among urban refugee youth. Specifically, our study aims to understand a) experiences regarding HIV testing and b) HIV self-testing perspectives, among urban refugee youth in Kampala.
Mohammadi, S, 2017, Experiences of inequitable care among Afghan mothers surviving near-miss morbidity in Tehran, Iran: a qualitative interview study	Iran	Afghan refugee woman	Qualitative, interviews	Barriers to health care access	This study explores experiences of maternal care among Afghan women surviving near-miss morbidity to increase insight into healthcare improvements for migrants.
Mukumbang, F. C, 2024, Experiences and perceptions of migrant populations in South Africa on COVID-19 immunization: an interpretative phenomenological analysis	South Africa	Asylum seekers, refugees from sub-Saharan Africa (Cameroon, DRC, Malawi, Nigeria, Rwanda, Somalia, Tanzania)	Qualitative, interviews	Barriers to health care access	We conducted a qualitative study to explore the experiences and perceptions of migrant populations in South Africa on COVID-19 vaccines to inform recommendations for improved COVID-19 immunization.
Munyaneza, Y, 2019, Challenges of women refugees in utilising reproductive health services in public health institutions in Durban, KwaZulu-Natal, South Africa	South Africa	African refugee women (Burundi, DRC, Rwanda)	Qualitative, interviews	Barriers to health care access	The aim of the study was to document the day-to-day experiences of women refugees and uncover their challenges regarding utilization of reproductive health services in public, institutions of Durban, KwaZulu-Natal.
Mutiso, V, 2019, Intrigues of Accessing Mental Health Services Among Urban Refugees Living in Kenya: The Case of Somali Refugees Living in Eastleigh, Nairobi	Kenya	Somali refugees	Qualitative, FGD	Barriers to health care access	This article explores the perceived mental-health-care access barriers affecting the resettled refugee population in Eastleigh, Kenya.

First author, Year, Title	Host country	Study population	Methods	Objectives short	Objectives
Nabulsi, D, 2020, Voices of the vulnerable: Exploring the livelihood strategies, coping mechanisms and their impact on food insecurity, health and access to healthcare among Syrian refugees in the Beqaa region of Lebanon	Lebanon	Syrian refugees	Qualitative, FGD	Barriers to health care access	This study aimed to examine the livelihoods, coping strategies, and access to healthcare among SR based on registration status and accompanying formal assistance.
Nara, R, 2020, A Multi-Methods Qualitative Study of the Delivery Care Experiences of Congolese Refugees in Uganda	Uganda	Refugee woman from DRC	Qualitative, interviews and FGD	Barriers to health care access	We aimed to explore the SRH experiences of Congolese refugees living in Uganda and ways that services could be improved.
Öztürk, G, 2024, Syrian refugees' experiences while receiving mental health services and psychiatric nursing care: A qualitative study	Turkey	Syrian refugees	Qualitative, FGD	Barriers to health care access	This study examined the experiences of Syrian refugees in a community center in Türkiye as they access mental health services and receive psychiatric nursing care.
Rustad, S, 2021, Maternal Health Care Among Refugees and Host Communities in Northern Uganda: Access, Quality, and Discrimination	Uganda	South Sudanese refugee mothers	Quantitative, questionnaire	Barriers to health care access and discrimination in health care	Our aim is to investigate whether mothers from the two groups (refugees and host population) experience the same access to and quality of maternal health services, and whether refugee- and host-community mothers perceive the maternal health services differently.
Stevenson, M, 2023, Qualitative assessment of the impacts of the COVID-19 pandemic on migration, access to healthcare, and social wellbeing among Venezuelan migrants and refugees in Colombia	Colombia	Venezuelan migrants and refugees	Qualitative, interviews and FGD	Barriers to health care access	As part of a larger study focused on HIV among Venezuelans residing in Colombia, qualitative interviews were conducted to understand this population's experiences and access to healthcare.
Tesfai, A, 2023, Human Rights Violations and Mistrust among Refugees in South Africa: Implications for Public Health during the COVID Pandemic	South Africa	Refugees, refugee community leaders	Qualitative, interviews	Barriers to health care access and discrimination in health care	In light of the suggestions that South Africa's COVID-19 response has increased healthcare discrimination and inequality for refugees, this paper explores refugees' experiences with the healthcare system during the COVID-19 pandemic, as well as how that influences their views on COVID-19 and COVID-19 prevention, including vaccination, using a lens of social trust.
Unternaher, J, 2024, Factors associated with attendance to and completion of prenatal care visits in Colombia among urban-residing Venezuelan refugee and migrant women	Colombia	Venezuelan refugee and migrant woman	Quantitative, questionnaire	Barriers to health care access	We aimed to assess uptake of and factors associated with prenatal care among Venezuelan refugees and migrants in Colombia.

First author, Year, Title	Host country	Study population	Methods	Objectives short	Objectives
Warkentin, T, 2024, Disability-Related Risks Among Women and Girls Who Are Forcibly Displaced from Venezuela	Brazil, Ecuador, Peru	Forcibly displaced girls and woman with disabilities from Venezuela	Qualitative, interviews	Barriers to health care access	In 2010, the United Nations Refugee Agency (UNRA) urged the United Nations to prioritize anti-discrimination and humanitarian support for refugees and migrants with disabilities. The UNRA has also highlighted the need for accurate data collection regarding refugee and migrant access to disability-related services. Having a comprehensive understanding of disability-related risks among Venezuelan refugees and migrants is critical to guide humanitarian programs and policies in the midst of this ongoing crisis. Our study aimed to address this need while maintaining the personhood of individuals with disabilities by using narrative storytelling to identify disability-related risks of women and girls during forced displacement from Venezuela.
Zaman, M, 2024, Experiences of pregnant Venezuelan migrants/refugees in Brazil, Ecuador and Peru: a qualitative analysis	Brazil, Ecuador, Peru	Pregnant Venezuelan migrants and refugees	Qualitative, micronarratives	Barriers to health care access	Our aim is to identify the challenges (including safety, accessible health care, and financial hardship, among others) faced by pregnant migrants/refugees in order to inform programming, services, and policies that will address their unique needs.
Zambrano-Barragán, P, 2021, The impact of COVID-19 on Venezuelan migrants' access to health: A qualitative study in Colombian and Peruvian cities	Colombia, Peru	Venezuelan forced migrants	Qualitative, interviews and life histories	Barriers to health care access	This research seeks to understand how COVID-19 has affected access to healthcare among migrants in Latin American cities.
Zihindula, G, 2017, Lived Experiences of Democratic Republic of Congo Refugees facing Medical Xenophobia in Durban, South Africa	South Africa	DRC refugees	Qualitative, interviews	Discrimination in healthcare	This research was undertaken to explore experiences of xenophobia by refugees from the Democratic Republic of Congo (DRC) with the health care system in Durban, South Africa.
