# Supplementary material for: Discrimination in medical settings as perceived by forcibly displaced people within low- and middle-income countries: a scoping literature review
Source: Public Health Rev. 2026 Oct 5;47:1609595. doi: 10.3389/phrs.2026.1609595 (PMC13677560; doi:10.3389/phrs.2026.1609595)
Supplement: Supplementary file 3 [file DataSheet3.PDF]

Supplementary File 3 – Table of Included Studies (Findings and Recommendations)

Table: Overview of studies included in review with main findings and conclusions / recommendations

Author, Year, Title	Findings	Conclusions / Recommendations
Arnold, C, 2014, A qualitative exploration of access to urban migrant healthcare in Nairobi, Kenya	(…) [The] lack of available drugs, primarily at public facilities was reported by government representatives, service providers, Kenyans and migrants alike. Service providers and several Kenyans commented that 'clients are given whatever is there' and 'usually they don't have enough', or that medication was 'very hard to get'. On the other hand, migrants appeared to interpret lack of drug provision as directly related to their immigration status, without acknowledging a widespread shortage. (...) Another participant described a second-hand account of discrimination that led to her reluctance to seek future services from that particular facility. It is unknown whether the healthcare providers in the scenarios described by participants intended to exhibit discriminatory behavior; however, the underlying motives do not change the outcome. Perceived discrimination led to failure to be treated and the subsequent avoidance of certain healthcare facilities. Not only might those individuals fail to access the same service in the future, but due to the ease of interpersonal communication within migrant communities, mistrust may be easily passed on to some or all of the local migrant community, causing more widely spread mistrust.	Despite articles from the 2010 Constitution of Kenya that assert the right to health for every person in Kenya, migrants continue to experience unique barriers in accessing healthcare. Efforts to eliminate these barriers should address policy-level interventions, strengthened networks and partnerships, improved migrant-sensitive services and especially continued research in migrant health. The inclusion of cultural competence training programs in government regulations and professional practice standards has been shown to effectively improve the abilities of healthcare providers to meet the needs of a diverse patient population and improve patient satisfaction (Beach et al., 2005).
Ay, M, 2016, The Perceived Barriers of Access to Health Care Among a Group of Non-camp Syrian Refugees in Jordan	Cognitive barriers were not common compared with other barriers. Discrimination by health personnel was the most frequently reported cognitive barrier (40.8%), and 29.1% of refugees did not trust the doctors or services provided. The high rate may be due to real discrimination or merely a misconception.	The capacities of health facilities at different levels should be increased. Enhanced information sharing among health providers can improve identification of needs and gaps. The capacities of health facilities need to be expanded to accommodate non-camp Syrian refugees, with international support to the ministry of health. To increase awareness of available services, mass SMS messaging may be used. Information sharing among all health care providers should be improved. Syrian Refugee Response Tracking, a web-based platform for better centralization, mapping, and analysis of humanitarian activities in Jordan, is an important initiative for information sharing.
Benavente, P, 2025, Deployment and uptake of COVID-19 vaccines for refugees and migrants in regular and irregular situations: a mixed-method multicountry study	Nine per cent ((126/1378), 95% CI 7.7% to 10.8%) of the respondents witnessed discrimination in the vaccination process, with Pakistan showing the highest level. In this country, the discrimination included instances directed at the respondents themselves (10.6% (53/499), 95% CI 8.2% to 13.6%), as well as observations of discrimination towards other migrants (3.4% (17/499), 95% CI 2.1% to 5.4%) and the public (2.6% (13/499), 95% CI 1.5% to 4.4%). Conversely, in Rwanda, respondents witnessed the least discrimination (2.5% (5/205), 95% CI 1.0% to 5.7%). In all countries, most of the discrimination cases observed were related to the health center staff, security personnel and the local population. Among vaccinated respondents, 15.5% ((185/1194), 95% CI 13.5% to 17.7%) said they	Despite the high coverage of COVID-19 vaccines among refugees and migrants in the study countries, the process had significant barriers. Simple vaccination registration procedures, targeted campaigns, mobile vaccination teams for hard-to-reach and vulnerable groups and building trust in the host country authorities are pivotal for future and inclusive vaccine deployments. Future research should focus on identifying and addressing barriers such as long wait times, distance to vaccination sites and trust issues. Targeted strategies like simplified registration processes, targeted campaigns and mobile vaccination teams can enhance vaccine uptake. Building trust with host country authorities and healthcare providers is also crucial to improve vaccine acceptance among refugees and migrants.

Author, Year, Title	Findings	Conclusions / Recommendations
	had perceived differences in how they had been treated compared with most of the population. Overall, this proportion was higher among migrants in regular situations (21.2% (104/490), 95% CI 17.8% to 25.1%) than migrants in irregular situations and refugees (13.9% (53/381), 95% CI 10.8% to 17.8%; and 8.7% (28/323), 95% CI 6.0% to 12.3%, respectively) ($p<0.001$); however, in Pakistan, 50.0% (41/82) of vaccinated migrants in irregular situations reported experiencing this difference in treatment.	
Bridi, L, 2023, Attitudes toward dementia and cognitive aging among Syrian refugees resettled in Jordan: a qualitative study	[The] lack of institutional support also manifests as significant barrier to healthcare including lengthy commutes and discrimination from staff. Participants talked about being unable to afford healthcare visits and life-saving treatments. A group of participants in one focus group described how the inaccessible medications have forced them to develop a medicine exchange program to sustain their necessary treatments. Jordan should continue to expand and provide free mental health services for Syrian refugees as it has already outlined in its national mental health plan. Additionally, participants revealed experiences of discrimination within the healthcare system. This has previously been reported as a barrier to Jordanian healthcare, highlighting the need for anti-discrimination strategies such as staff training to reduce this strain on refugees.	Findings from this study are the first to examine the attitudes of Syrian refugees in Jordan toward dementia and cognitive aging. These results could provide essential data inclusive of refugees as Jordan develops its National Dementia Plan.<br>Recommendations: Investing in dementia awareness interventions and age-friendly neighborhoods may benefit aging refugees in limited-resources settings. Advance research to understand the cognitive needs of aging Syrian refugees. Reinforce awareness of the risk and protective factors of dementia. Invest in healthy/age-friendly housing and neighborhoods. Remove healthcare barriers to promote care-seeking behaviors. Expand assistance and funding to support basic living needs.
Chirau, T. J, 2024, Access to healthcare by undocumented Zimbabwean migrants in post-apartheid South Africa	Many migrants shared personal stories about being discriminated against. The majority of migrants reported that healthcare workers prioritized South African People compared to foreign nationals. Two sub-themes contributing to that were cited as the migrant status (undocumented) and nationality (country of origin).	Migrants continue to face challenges while accessing subsidized health care. This study confirms that medical xenophobia is generally present in the public health care centers, at least for the sampled undocumented Zimbabwean migrants. The majority of undocumented migrants cannot afford to pay for private healthcare. This study demonstrates the significance of standard operating procedures that agree with legal frameworks that can enable undocumented migrants to receive healthcare and provide guidance to healthcare providers on how to give quality and dignified care to migrant patients. Overall, the findings of this study, and other studies remain somewhat the same; no unified approach has been developed by the government to deal with the issue. This indicative of inefficiencies to resolve the impasse around access to health by undocumented migrants. Systemic and ongoing barriers to receiving public healthcare indicate that the South African administration has failed to meaningfully address the problem.
Crush, J, 2014, Medical Xenophobia and Zimbabwean Migrant Access to Public Health Services in South Africa	In South Africa, migrants seeking treatment are categorized and triaged, not on the basis of their health status, but their race, language and national origins. Some facilities operate two queues, one for locals and the other for foreign nationals. Everyone in the first queue is attended to before anyone in the second is seen. The healthcare provider thus builds into their triage assessment, a judgement on whether the patient is South African or not, speaks a local language or not, is foreigner or not, is wanted or not. A number of respondents noted the dramatic change in attitude that occurred when they were identified as Zimbabwean. Pregnant women are generally treated with a modicum of courtesy and respect at public health institutions. However, once their identity as Zimbabweans is	The research for this study clearly demonstrated that foreign migrants are also singled out for unethical and abusive treatment.<br>Such medical xenophobia categorizes patients by language, appearance and national origin and treats them accordingly. We certainly do not wish to claim that all health workers display the kinds of attitudes and behaviors described in this paper. The Southern African Clinicians Society, for example, has played a leading role in promoting equal treatment for all people living with HIV, foreign and local (SAHIVSOC Citation 2007). However, by conducting research in six different communities in two major cities, we have sought to move beyond existing studies that tend to focus on only one area or community. Importantly, the situation is

Author, Year, Title	Findings	Conclusions / Recommendations
	revealed, the quality of care immediately declines and some even report a torrent of verbal abuse questioning their right to bear children in South Africa and demanding that they return home. (...) This study has shown that Zimbabwean migrants continue to be denied treatment on the grounds that they are not South African or cannot show the 'correct' documentation (correctness being variously and inconsistently defined). Even those with such documentation face verbal and physical abuse while undergoing treatment, especially from frontline health workers including nurses. Undocumented migrants tend to avoid public clinics and hospitals altogether. Not only are they unlikely to be treated, they also run the risk of being arrested and deported by the police.	described only from the point of view and through the experiences of migrants rather than healthcare providers. Additional research is therefore required, if only to understand the health providers' justification for and rationalization of attitudes and behaviors that are patently xenophobic in nature.
Dadras, O, 2020, "It is good, but I can't afford it …" potential barriers to adequate prenatal care among Afghan women in Iran: a qualitative study in South Tehran	Some woman had bitter experiences or experienced discrimination. Some participants did not like to be questioned about their nationality because they perceived that as discrimination. However, it may be the tone or language used when being asked about their nationality which led to participant's mistaken perception that they were being discriminated against. (...)	The findings of present study emphasize the necessity of available and most importantly, affordable prenatal care for Afghan women in Iran. Providing an affordable health insurance with adequate coverage of prenatal and delivery services, could reduce the financial burden, facilitate the access, and ensure the maternal and child health in this vulnerable population. The issues of fear and concern of deportation must be removed for at least illegal Afghan mothers to ensure their access to maternity care and improve the health of both mother and offspring. The findings emphasized the necessity of available and most importantly, affordable prenatal care for Afghan women in Iran. Providing an affordable health insurance with adequate coverage of prenatal care services, could reduce the financial burden, facilitate the access to prenatal care, and ensure the health of mothers and offspring among this vulnerable population. Apparently, the situation for illegal Afghan migrants and refugees is more critical and should be considered by international agencies and regional health authorities where this population predominantly reside. The issues of fear and concern of deportation, at least, must be removed for the illegal Afghan mothers to ensure the safety and optimal health of both mother and child. Further studies are of critical necessity to explore the issues surfaced themselves in present study and to determine the prevalence and distribution of such issues and further find solutions to address them in future national policy and guidelines. [Further] we emphasized the necessity of interventions to enhance the intercultural-competency of health personnel delivering health services at Afghan-concentrated communities in Iran.

Author, Year, Title	Findings	Conclusions / Recommendations
Dahlberg, M, 2023, Conceptualizing xenophobia as structural violence in the lives of refugee women in Gauteng, South Africa	Although the women interviewed in this study had the right to health care, xenophobia prevented them from getting the right treatment and medicine. At the hospitals and clinics, even the ability to speak English does not help the respondents to receive health care, as the nurses and people working in the reception often speak to them in South African languages such as Zulu or Sotho. In most cases, they do not get assistance. Instead, they are told to sit down and wait, to come back another day, ignored or told that the hospital does not have the medicine that they need.	Our attempt in this article is to understand the embeddedness of xenophobia in institutions and the ways in which these structural inequalities inform and shape the everyday experiences and lives of refugee women. While keeping an empirical grip on experiential narratives on xenophobia, we draw attention to the macro structures that shape everyday experiences of violence for refugee women. Three main ideas associated with structural violence are identified: unequal access to resources, constrained agency and dehumanization. We are not arguing that every refugee woman living in Gauteng experiences the same vulnerabilities as the women in this study. However, little in these stories is unique. The structural barriers that (the) women in this study meet, highlight forces that constrain the life of many refugee women, even beyond South Africa. To approach an end to these violations, we need to identify the forces that create structural violence, and critically assess the structures that keep some people on the margins.
Doğan, N, 2019, Syrian refugees' experiences with mental health services in Turkey: "I felt lonely because I wasn't able to speak to anyone"	Some participants sometimes felt they were treated differently from Turkish citizens because they were refugees. They said that they felt this way more often in other hospitals, but less when an Arabic-speaking doctor was available.	Nurses should be aware of the barriers experienced by refugees that affect the quality and accessibility of psychiatric services.
Genç, H. D, 2024, Migrants' access to healthcare services: evidence from fieldwork in Turkey	Our findings revealed a decline in acceptability, especially for Syrian refugees. Among all interviewees, almost all Syrian refugees shared experiences of encountering discrimination when seeking healthcare. During research, Syrian refugees reported facing mistreatment and obstacles in accessing healthcare, primarily due to their Syrian identity. These experiences were rather attributed to nurses, other hospital staff, and even Turkish patients. Instances were shared where hospital staff and patients united in expressing negative sentiments towards Syrian refugees, leading to verbal abuse and marginalization within hospital waiting rooms. The forms of discrimination varied widely, ranging from refusal to give information in the hospitals to physical violence. The experiences of Syrian women in accessing reproductive health, particularly during pregnancy and childbirth, evolved beyond discrimination. Given that Syrian refugees were already in a precarious position, the discrimination and mistreatment they experienced led many of them to avoid seeking care until their health situation reached a critical stage. They visited health institutions only when they had a pressing problem, such as acute appendicitis, unbearable tooth pain, or excessive bleeding. One interviewee stated that she would only go to the hospital "while she was dying." Referring to *acceptability*, Syrian refugees explained that they opted for both migrant health centers and informal clinics to avoid mistreatment, discrimination, and hostility when seeking care. Among all interviewees, almost all Syrian refugees shared experiences of encountering discrimination when seeking healthcare.	In concluding, we emphasize that Turkey's ministry of health (MoH) needs to take effective steps to prepare itself to meet the needs of the more diverse population that is in the making. Though there seems to be at least a mediocre fit between migrant patients and the Turkish health system, migrants still encounter various barriers in accessing healthcare and are sometimes exposed to discrimination and abuses in the system. Following the reforms launched in 2003, the MoH's role in the provision of health services has decreased, but migrants' experiences in accessing healthcare in Turkey reveal the necessity for the institution to adopt a strengthened role in regulating service provision and financing, including insurance and direct payments. Moreover, it should actively monitor and regulate marketization and commodification processes in the health system while safeguarding vulnerable patients, including migrants.

Author, Year, Title	Findings	Conclusions / Recommendations
Kabakian-Khasholian, T, 2022, "A person who does not have money does not enter": a qualitative study on refugee women's experiences of respectful maternity care	Most of the participants reported problematic verbal and non-verbal interactions with the healthcare providers. They labelled most of the verbal communication with the hospital staff as disrespectful, discriminatory, and insulting. They spoke of several instances when they felt discriminated against and subject to verbal abuse and physical violence. They described how they found themselves in conflicts with healthcare providers with disrespectful attitudes who did not empathize with women's feelings and prevented them from expressing their pain. Throughout these interactions, women were being blamed for expressing pain, bearing many children and for expressing their needs of holding their newborn. This process of communication nurtures feelings of guilt and being seen as an outsider.	This study shows how disrespect and mistreatment are intertwined in a complex system which is devised to ensure access to facility-based birth for displaced populations. Implications for programs and policies point to the need for strengthening capacity and for providing resources for the adaptation of global guidelines into context-specific strategies for the provision of quality maternity care during humanitarian crises and beyond.
Karaki, F. M, 2021, Noncommunicable Disease and Health Care-Seeking Behavior Among Urban Camp-Dwelling Syrian Refugees in Lebanon: A Preliminary Investigation	Some Syrian Refugees cited negative treatment by certain health care workers at specific health facilities as a factor that influenced their access to care. Some described their own negative interactions, while others witnessed or heard of these incidents second-hand. In all cases Syrian Refugees decided not to return to the facility.	In this population, we found a higher prevalence of NCDs at younger ages than in the general Syrian refugee population. We described perceived barriers to accessing health care, including the noteworthy finding of perceived discrimination by health care workers in a linguistically and culturally related host community. We discussed social determinants of health within the camp and refugees' ability to access health care services. Future research should examine correlations between specific living conditions and increased prevalence of NCDs. Additionally, potential future interventions should focus on mechanisms to decrease cost and improve relationships between refugees and health care workers to improve refugee access to care. After establishing a sufficient evidence base regarding disease prevalence and needs assessment, focus can be shifted to evaluating interventions and effective public health prevention measures. Understanding how different programs operate within the camp, critically evaluating performance, and systematically incorporating effective care models into other areas will lead to long-term, evidence-driven interventions that will improve clinical outcomes among refugees.
Khalifeh, R, 2023, Experiences of Cultural Differences, Discrimination, and Healthcare Access of Displaced Syrians (DS) in Lebanon: A Qualitative Study	Following the interviews, DS reported being rejected and/or discriminated against while receiving healthcare services. The stereotyped behavior of health professionals induced ambivalent feelings among DS, thus leading to them discontinuing the care. Sometimes, DS avoided coming back to the center because of this perceived feeling of rejection. They said that health professionals speak to them in a threatening and aggressive way and sometimes even leave them waiting without a valid reason. For their part, the displaced Syrians, following the interviews, expressed themselves regarding the rejection they felt from Lebanese health professionals, which constitutes a barrier in the provision of care. The stereotyped behavior of health professionals induces ambivalent feelings among displaced Syrians and thus leads to a halt in the continuity of care.	The number of displaced Syrians in Lebanon is roughly equal to a quarter of its citizens; there is an urging need to restore the Lebanese health system to ensure the equitable provision of health services for displaced Syrians and appropriate working conditions for health professionals.

Author, Year, Title	Findings	Conclusions / Recommendations
Khalifeh, R, 2023, Healthcare bias and health inequalities towards displaced Syrians (DS) in Lebanon: a qualitative study	DS reported experiencing different forms of biases in the Lebanese system. Discrimination and prejudice are revealed through difficulty in accessing health services, refusal of hospital admission, and extended waiting time. Claims such as "You cannot be admitted to hospital because we do not have additional places for Syrians" are interpreted as a form of hatred toward Syrians, which is also a form of health inequity. DS claimed they are not getting the same quality health services as Lebanese. This discrimination was revealed in the DS' perception through the inappropriate ways health care providers communicate with them. Displaced Syrians confirmed that the main issue of accessing health services is discrimination. They considered they were the victims of hostility, lack of respect, and prejudice. They claimed that they felt ignored and neglected and that their treatment was inappropriate. They also denounced carelessness in the way health care providers interacted with them. This appears to be connected with health care providers' inadequate communication with the displaced Syrian due to their discriminating and stereotypical viewpoints. It is important to emphasize that the DS perception of discrimination is not linked to health care providers lacking skills but rather to a particular situation explained by the occurrence of several personal, contextual, and geographical factors leading to health inequalities toward displaced Syrian in Lebanon during the provision of care.	Based on the perceptions and experiences reported by participants, the underlying causes of biases are due to the fragility of the Lebanese healthcare system when facing a humanitarian crisis as well as a collapsing infrastructure torn by past wars and the current socio-political and financial crises in the country. Global initiatives are required to provide the necessary resources needed for offering equitable health services. Such initiatives involve addressing biases, health inequities, discrimination, and the lack of a Lebanese infrastructure system for the provision of healthcare. Addressing health inequalities remains a major health objective in achieving health equity on the micro level (cultural awareness and competencies) and macro level (equitable distribution of resources, implementation of a universal health coverage) in order to guarantee quality healthcare services to DS.
Kibiribiri, E, 2016, Exploring disparities in prenatal care between refugees and local South African women	There was a general agreement that refugees only attended a particular health facility because they resided in that specific area. 39.2% of refugee woman stated that they would not return to the same facility if pregnant again. Staff attitude and neglect were the most common reasons for refugees stating that they would not recommend the facility (34.5% each). Other common factors that made the refugees dislike the health facility were reported health care workers xenophobia (43.6%), carelessness (42.3%), and inability to communicate with refugees (37.2%). Refugees who were dissatisfied with the service would not return to the clinic for reasons collectively described as the perceived xenophobic attitude of health care providers. The language barrier was highlighted as a key impediment to engaging in healthcare dialogue; this barrier, coupled with the working conditions in an already overburdened health facility, leads to health care providers appearing to be discriminatory and intolerant.	There were no significant disparities in prenatal care; however, refugees unable to communicate in the local languages reported that they were not provided with relevant health information and occasionally faced restricted access to prenatal services. The required prenatal services should be delivered to all pregnant women, including refugees, through the identification of trained interpreters among existing health care providers to assist with history taking, education, and provision of information to patients who do not understand the local language. Alternatively, community members could be trained to assist with interpretation. Pamphlets on healthcare services should be written in more than one language and in languages most commonly understood by refugees attending the health facility. A course in cultural diversity could assist clinical staff to understand and develop tolerance toward refugees. Further, health association bodies (e.g. the South African Nursing Council) should be encouraged to promote training and registration of foreign nurses. Finally, in the absence of data for maternal health outcomes among refugees in South Africa, it is strongly recommended that this be explored in future Confidential Enquiries into Maternal Deaths.
Logie, C. H, 2021, Intersecting stigma and HIV testing practices among urban refugee adolescents and youth in Kampala, Uganda: qualitative findings	Participants reported enacted and anticipated stigma experiences when interacting with healthcare providers, including stigma associated with being a refugee, a young person, and/or a sex worker. Participants also experienced enacted stigma when testing for HIV that aligns with the aforementioned perceived stigma towards sexually active youth: "Even some health workers begin blaming you for getting HIV without knowing the whole story of how you got HIV. They start asking that	Intersecting stigma rooted in fear, misinformation, blame and shame, legal precarity and healthcare mistreatment constrain current HIV testing strategies with urban refugee youth. Findings align with the Health Stigma and Discrimination Framework that conceptualizes stigma drivers and facilitators that devalue intersecting health conditions and social identities. Findings can inform multi-level

Author, Year, Title	Findings	Conclusions / Recommendations
	'at this young age you are HIV positive?' This was also expressed among young refugee men, who explained how healthcare workers assumed they engaged with sex workers. Anticipated stigma was also rooted in confidentiality concerns where participants felt that healthcare workers were more likely to share HIV test results and breach confidentiality with refugees. These confidentiality concerns may result in seeking HIV testing in a different community. Others discussed mistrust due to past experiences of being tested for HIV without consent.	strategies to foster enabling HIV testing environments with urban refugee youth, including tackling intersecting stigma and leveraging refugee youth peer support.
Mohammadi, S, 2017, Experiences of inequitable care among Afghan mothers surviving near-miss morbidity in Tehran, Iran: a qualitative interview study	Many interviewees felt that they had been subjected to discrimination at hospitals. Inequitable health policies and degrading treatment in combination with socioeconomic disadvantages appeared to shape a perception of dissociation from the Iranian health system and society among our participants. Our participants mainly experienced mistreatment in the form of being subject to discrimination and insufficient medical attention in the course of receiving maternal care. Verbal abuse was also cited. One woman thought that obstetric professionals behaved as though Afghans were stupid. She recalled the popular sentence that Afghans heard: 'You are Afghanis; if your baby dies, you'll come back next year with another'. One woman recalled memories of her first childbirth at a maternity center, where she heard midwives say that they were unable to put up with Afghans any longer. Participants generally mentioned that they were not informed about what had happened to them at the hospital. They thought doctors did not care to explain anything to them. Mothers had inadequate insights about the complications they developed and the reasons they were delivered by CS even after their successful recovery and discharge from the hospital. These kinds of experiences broke the necessary trust between patients and professionals and appeared to affect care-seeking in the future.<br>A few women, however, were satisfied with the care services they received and expressed their thankfulness to God for being alive after experiencing all of those complications. The challenge for the health system and professionals is to provide equitable care with dignity and improve communication skills with caring attitudes toward ethnic minorities. Antenatal visits provide the best and most appropriate opportunities to tackle health illiteracy in Afghan women.	Near-miss survivors' experiences provided remarkable insights into maternal care of Afghans in Iran. The challenge for the health system and professionals is to provide equitable care with dignity and improve communication skills with caring attitudes toward ethnic minorities. Antenatal visits provide the best and most appropriate opportunities to tackle health illiteracy in Afghan women. This study highlights various factors that contributed to delays and disparity in the maternal care provided to Afghan migrants. Our results explored the negative experiences of being subjected to discriminatory care and receiving degrading treatment in Tehran. Therefore, it is essential for obstetric professionals to be aware of how mothers experience the care they provide. Moreover, adopting a rights-based approach in training and in monitoring maternity services may ensure effective, respectful, and equitable care. Migrants affected by socioeconomic and humanitarian challenges and the special needs of this minor population, such as health literacy, should be given additional attention within antenatal consultations. To improve migrants' maternal outcomes, policy makers should consider barriers to achieving equitable care and should include maternal voices when developing and structuring health programs.
Mukumbang, F. C, 2024, Experiences and perceptions of migrant populations in South Africa on COVID-19 immunization: an interpretative phenomenological analysis	The study participants believed that migrants were being excluded from accessing COVID-19 vaccines. One participant perceived discrimination at one of the COVID-19 vaccination centers. "But some nurses said you guys want to get all our vaccines. Go back to your countries and get vaccinated… You can see that segregation, saying, 'No, you're not a South African.' So, I was uncomfortable going for the second jab because of those statements." While others felt that they, as migrants, were being excluded from the national COVID-19 immunization program at various levels, others conceded that migrants were not being excluded based on their experiences and observations.	COVID-19 vaccine inclusiveness, awareness, and uptake should be enhanced through migrant-aware policies and actions such as community mobilization, healthcare professional training, non-monetary incentives and mass media campaigns.

Author, Year, Title	Findings	Conclusions / Recommendations
Munyaneza, Y, 2019, Challenges of women refugees in utilising reproductive health services in public health institutions in Durban, KwaZulu-Natal, South Africa	Xenophobia is the most serious barrier to healthcare access for refugees. In this study, the findings revealed that all participants experienced xenophobia and discrimination in public healthcare facilities while seeking reproductive health services. They reported facing negative attitudes from healthcare professionals, mostly nurses, because of the fact that they are non-citizens. The study also revealed that in some instances the participants were neglected and others denied treatment because they were foreigners.	The findings revealed that women refugees in Durban, KwaZulu-Natal, face many challenges such as medical xenophobia and discrimination in their attempt to seek reproductive health services in public healthcare facilities, making them even more vulnerable. Assisting women refugees with their reproductive health needs will remediate the challenges they face. Based on the findings, recommendations were made to the Department of Health regarding the training of healthcare workers in handling refugees' issues, specifically women in this scenario, in customer care, as well as in the refugee rights to accessing and utilising healthcare services in South Africa. Creating awareness about women refugees' problems and concerns in relation to reproductive health services will also improve the healthcare providers' attitudes towards refugees. The Health Department recommended considering either providing an interpreter at facilities most visited by refugees or employing one of the refugees trained and qualified to offer such services, to accommodate refugees in different languages.
Mutiso, V, 2019, Intrigues of Accessing Mental Health Services Among Urban Refugees Living in Kenya: The Case of Somali Refugees Living in Eastleigh, Nairobi	There was also perceived stigmatization of Somali refugees by the healthcare providers, as one participant reported: "Even if I have a mental condition, I do not know whether I can trust the doctor because, the people at the community in Eastleigh have shown me a lot of stigma and discrimination in various service deliveries including health care" (Male adolescent FGD respondent in Eastleigh).	This study recommends blending Somalis' indigenous treatment methods with Western methods in order to increase the cultural competency of the spectrum of mental help available to refugees. Moreover, there is need for the Kenyan government and international agencies working with refugees to ensure that mental health services are packaged as part of humanitarian assistance provided to refugees because of their vulnerability to developing psychological problems. Additionally, state and nonstate actors on refugee matters in Kenya should ensure that all urban refugees have the necessary supporting social amenities, such as for addressing the legality issues and improving mobility to prevent new-onset psychological adversity.
Nabulsi, D, 2020, Voices of the vulnerable: Exploring the livelihood strategies, coping mechanisms and their impact on food insecurity, health and access to healthcare among Syrian refugees in the Beqaa region of Lebanon	Many registered and unregistered participants stated that discrimination and poor treatment in hospitals by healthcare professionals were also a common experience: "*When I went to get the operation*, *they were yelling at me*, *and when I was done*, *they lifted me and then threw me on the bed*, *and even under anesthesia you feel the pain*, *you feel the pain of being thrown on the bed*" (W.R.A.1. P5).	Our findings shed light on the economic and health disparities among marginalized Syrian Refugees, with the lack of registration and formal assistance increasing their vulnerability. More tailored and sustainable humanitarian programs are needed to target the most vulnerable and hard-to-reach groups. These findings also call for integrated and sustainable humanitarian programs that address the basic food and healthcare needs of refugees while strengthening the resilience of healthcare systems in host countries. Given the protracted nature of the Syrian refugee crisis, a paradigm shift is also needed to address the social determinants of health among refugees, including the protection and legal status of refugees together with policies that can consider the temporary integration of refugees into the workforce. These policies can facilitate the access of refugees to safe and dignified sources of livelihoods, which in turn can reduce their economic vulnerability and help improve their food security levels and health status. In addition, future research is needed to examine potential interventions and strategies that aim at reaching the hard-to-reach groups and those most marginalized groups. With the ever-growing complexities and challenges faced by the forcibly displaced populations and their host countries, humanitarians, policy makers and researchers will need to work

Author, Year, Title	Findings	Conclusions / Recommendations
		collectively to design and evaluate sustainable solutions that can promote resilience among these vulnerable communities.
Nara, R, 2020, A Multi-Methods Qualitative Study of the Delivery Care Experiences of Congolese Refugees in Uganda	The Congolese women in our study repeatedly reported experiencing discrimination when seeking care. As Marie, a 33-year old refugee living in Kampala explained, "You go to the hospital and you have [an] emergency case, but they will not mind. Sometimes they will ask you for money and you don't have it. Or sometimes they will say, 'Ah, leave that Congolese'. When they hear you are a Congolese, they always increase the price." That many refugee women are unable to communicate effectively with their health service professionals overlaps with the issue of discrimination.	Efforts to increase trained healthcare staff, improve supply-chain management, and maintain infrastructure and equipment are imperative. Ensuring compliance with anti-bribery and anti-corruption policies and supporting respectful maternity care is also important. Creating approaches to overcome language barriers is crucial to minimizing miscommunication and building patient-provider trust.
Öztürk, G, 2024, Syrian refugees' experiences while receiving mental health services and psychiatric nursing care: A qualitative study	Most participants experienced discrimination and stigmatization while trying to receive mental health services. Although some individuals claimed that they did not encounter any racism, the majority of the participants stated that less time was allocated to them because of their race, their waiting times were longer, meetings were kept short, citizens of the country were given priority, and the attention shown to citizens was not applied to them. Some individuals also stated that they avoided getting health services because of these negative experiences. Furthermore, the participants stated that they felt worthless because they were excluded due to their race.	This study determined that Syrian refugees face barriers in accessing and receiving mental health services. They stated that mental health professionals in Türkiye approach them with empathy, particularly those in psychiatric nursing. Healthcare professionals may be trained in culturally sensitive care to increase awareness.
Rustad, S, 2021, Maternal Health Care Among Refugees and Host Communities in Northern Uganda: Access, Quality, and Discrimination	We see the risk of feeling discriminated against during ante natal care increases from 4% among the host population to 10% among the refugee women[1]. Mothers who answered that they were treated worse or much worse were also asked why they felt this way. Among the 29 refugee mothers who answer this way, 20 responded it was due to their refugee status or language. For giving birth, 18 out of the 29 who felt treated worse mentioned the same reasons. Among the host community mothers, the reasons for feeling treated worse were more diverse.	The way women feel treated at the health facility during maternal health care is an important aspect of quality care. While there seems to be equal access to resources between refugees and host community mothers in Northern Uganda in terms of access to and quality of care, there is still a discrepancy between the two groups in terms of how the women feel treated. Policymakers and practitioners in the health sector should pay attention to these perceived inequalities between refugees and women from the host communities to ensure equally inclusive treatment across groups. Future research should try to devise more robust, nuanced measures that distinguish between the objective quality of medical institutions, personnel, and care.

[1] Abstract: Refugee mothers are also 4% points more likely to feel discriminated against during ANC compared to their counterparts in the host community (p-value < 0.05).

Author, Year, Title	Findings	Conclusions / Recommendations
Stevenson, M, 2023, Qualitative assessment of the impacts of the COVID-19 pandemic on migration, access to healthcare, and social wellbeing among Venezuelan migrants and refugees in Colombia	Venezuelans with irregular migration status are able to access emergency services through the Colombian health security system, however participants reported inconsistent and discriminatory practices in determining what qualifies as an emergency. One key informant expressed that "*you have to, to put it bluntly, have your intestines hanging out, so that they will attend to you*" (KI3, 47, male)	Colombia has enacted increasingly inclusive migration policies for Venezuelan refugees and migrants within the country; findings from this study underscore the necessity for such policies both in and outside of the Colombian context.
Tesfai, A, 2023, Human Rights Violations and Mistrust among Refugees in South Africa: Implications for Public Health during the COVID Pandemic	The participants described disturbing experiences of encounters with healthcare workers among members of their community, particularly negative encounters with nurses. Interestingly, refugees reported having much better experiences when encountering doctors for tertiary care. These negative experiences meant that refugees typically avoided going to public healthcare facilities. Experiences of discrimination were mostly shared about or by women, who had more contact with public healthcare facilities because they needed reproductive services. Refugees were accused of draining resources that are meant only for South Africans. The pressures of COVID-19 limited healthcare for everyone in South Africa, but as these findings reflect, pandemic responses in South Africa exacerbated discrimination and abuse faced by refugee communities, who already faced a number of barriers to healthcare access, even before COVID-19. This pattern of exclusion, at both the interpersonal and institutional levels, contributed to refugees avoiding seeking care or services, even when they were available. The findings of this study suggest that discrimination itself is a pandemic for refugees in South Africa, and the COVID-19 pandemic was just another opportunity for its expression. These sets of experiences of discrimination among refugees represent the structural discrimination vulnerable groups experience in public healthcare due to systemic issues that impact access and the quality of healthcare.	Our results explored how mistrust threatens the health of the refugee community by limiting access to the prevention and treatment of COVID-19. However, this also impacts the potential of various public health initiatives and responses intended to mitigate COVID-19 countrywide because public health during a pandemic requires whole-population initiatives to mitigate disease spread. Overlooking refugees' needs for information and services in the pandemic response implies that, at best, refugees are not seen as belonging to the South African community; however, at worst, oversights and exclusions can be seen as aligning with systemic goals, benefitting a strained healthcare system in the short term, and bolstering stated governmental goals of encouraging refugees' departure from South Africa overall. Thus, the mistrust identified in refugee communities in our sample could be seen as a justified response to the systemic and social barriers they experience in the healthcare system.
Unternaher, J, 2024, Factors associated with attendance to and completion of prenatal care visits in Colombia among urban-residing Venezuelan refugee and migrant women	Over 20% of total participants reported being treated with empathy in prenatal care because of Venezuelan origin; however, this was reported by more participants who had completed <4 prenatal care visits than those who completed more (24.7% vs. 19.0%). We found that 12–15% of participants reported experiences of or witnessed discrimination on the basis of one's migration history or country of origin at across our discrimination measures. These were different across participants by number of prenatal care visits, as women who had received <4 visits were more likely to report past experiences of or witness of denied prenatal care or disrespectful treatment during prenatal care, compared to women who had received ≥4 visits. However, those who had experienced denial of prenatal care had a 42.8% lower likelihood of completing at least four prenatal care visits, which underscores the potential impact of discrimination in future attempts to access to what should be widely available prenatal care for Venezuelan refugees and migrants.	Prenatal care attendance is suboptimal among Venezuelan refugees and migrants, particularly those with an irregular migration status, despite that prenatal care became officially available in 2018 to all Venezuelans in Colombia regardless of migration status. Reducing barriers to prenatal care by ensuring Venezuelan refugees and migrants are aware of available care, are supported in navigating the health system, and by preventing discrimination and stigma in the health facility are critical to ensuring the health and wellbeing of displaced people, their children, and the surrounding community. Our findings suggest that Venezuelan refugees and migrants, particularly those with an irregular status, had suboptimal prenatal care attendance even after policy change granted access to care in 2018. The barriers to accessing healthcare faced by Venezuelan refugees and migrants may overlap with those experienced by some Colombians, particularly those who are uninsured and/or low-income, live in remote rural areas, or who are from minoritized communities. They may also experience unique barriers related to

Author, Year, Title	Findings	Conclusions / Recommendations
		awareness of rights to prenatal care and processes to access that care, including their own awareness as well as awareness among healthcare providers. Confusion related to which types of healthcare are available to people with an irregular status, or the Venezuelan community broadly, may be alleviated by more recent policy changes. To further avert suboptimal prenatal care attendance, integrating migrants and refugees into the healthcare system and developing comprehensive communication campaigns may assist Venezuelans with adapting to a different healthcare system, potentially reducing downstream costs associated with worse postnatal and neonatal outcomes.
Warkentin, T, 2024, Disability-Related Risks Among Women and Girls Who Are Forcibly Displaced from Venezuela	The final theme that emerged was disability-related experiences in healthcare. Women and girls with disabilities had both positive and negative experiences obtaining healthcare in host countries. Specifically, some people were readily able to book medical appointments and obtain necessary medications and surgeries for both acute and chronic medical conditions (N = 16), In contrast, several narratives (N = 11) described long wait times in host countries that precluded rapid access to necessary medical care. Finally, two narratives described poor healthcare experiences whereby discrimination limited the provision of optimal care. Discrimination was identified as an attitudinal barrier whereby women with injuries caused by violence were refused optimal care. Once again, gender, refugee and disability status intersect within healthcare, creating disproportionate oppression requiring directed humanitarian assistance to ensure individuals receive the care they need. Venezuelan refugees and migrants with disabilities described experiences of discrimination, violence, and physical challenges, such as exacerbation of symptoms while in transit. In host countries, refugees and migrants experienced a lack of disability-related accommodations in the workplace and long wait times when trying to obtain healthcare.	In host countries, refugees and migrants experienced a lack of disability-related accommodations in the workplace and long wait times when trying to obtain healthcare. Since discrimination is a cross-cutting issue, human rights awareness highlighting the dignity of persons with disabilities is imperative. Resources and support for Venezuelan refugee and migrant women and girls with disabilities should aim to create accessible employment opportunities, safe and timely access to medical care, and prioritize violence prevention. Based on our results, the following recommendations can be made: (1) Host countries should empower persons with disabilities and strengthen state institutions in the observation of respect for those with disabilities so that they may exercise their rights with dignity. This target should include the development of accessible employment opportunities and the promotion of timely access to medical care. (2) NGOs and governments, in their intersectional approaches, should include families of persons with disabilities. Response programs should be targeted towards identifying and appropriately addressing the individual needs of each person with a disability. (3) Specific programming required includes violence prevention and safety for women and girls with disabilities. Further, transit systems and shelters should be adapted for different types of disabilities. Future research should consider examining the unique needs of subpopulations of refugees and migrants with disabilities, given that persons with disabilities represent a broad, heterogeneous group whose individual needs may vary.
Zaman, M, 2024, Experiences of pregnant Venezuelan migrants/refugees in Brazil, Ecuador and Peru: a qualitative analysis	There were many instances of xenophobia and humiliation described in the micronarratives. In fact, many women experienced xenophobic behavior from health- care workers such as racial slurs and had trouble accessing adequate pregnancy care upon arrival to their destination countries. Pregnant women were treated badly in healthcare settings because the staff and administration sometimes shared beliefs that all Venezuelan women were only in their country to give birth to their children and thereby claim citizenship. They are often referred to as "Veneca" which is a racial slur against Venezuelans. Participants also described experiences where healthcare staff treated them with hostility. The xenophobia experienced from healthcare workers sometimes put women at risk of life-threatening situations, exacerbated their vulnerability, and affected the quality of health care	Pregnant Venezuelan migrants/refugees are a vulnerable population that encounter complex gender-based and societal issues that are rarely sufficiently reported. The findings of this study can inform governments, non-governmental organizations, and international organizations to improve support systems for pregnant migrants/refugees. Based on the results of our study we recommend addressing xenophobia in health-care centers and the lack of shelter and food in host countries at various levels, creating support spaces for pregnant women who experience trauma or violence, and connecting women with reliable employment opportunities and maternal healthcare. We recommend the following to governments, as well as to non-governmental, international and social support organizations:<br>1. Address xenophobia at health-care centers in host countries. This would include

Author, Year, Title	Findings	Conclusions / Recommendations
	received. Many narratives described medical negligence which risked harm to their child and themselves. A common theme of distressing encounters with healthcare professionals marked by derogatory comments and discriminatory attitudes emerged from the micronarratives. The stories also illustrate the harmful stereotypes present in host countries which can result in refusal of care and negligence. These experiences compromise the emotional wellbeing of women and also create a significant barrier to women accessing the necessary maternal healthcare they need.	development of policies, training, and regulation, in collaboration with health-care workers, administrative staff and policy makers.<br>2. Create support spaces for pregnant women who have experienced xenophobia or gender-based violence.<br>3. Find additional and alternative ways to address the lack of shelter, food and water for migrants/refugees, particularly pregnant women, when they arrive at their host country.<br>4. Include pregnant women –and their specific needs– in regularization and socioeconomic integration initiatives, prioritizing safe, appropriate and reliable livelihood strategies.<br>5. Provide access to contraception and sexual health resources, including prenatal and postpartum care.<br>6. Promote solidarity and empathy within the healthcare system, as well as other public and private spaces. Public policies should be more inclusive and recognize the potential of refugees/migrants to contribute to their host community if provided with appropriate assistance and support.<br>Solutions implemented by organizations should be developed in partnership with and tailored specifically for pregnant women.
Zambrano-Barragán, P, 2021, The impact of COVID-19 on Venezuelan migrants' access to health: A qualitative study in Colombian and Peruvian cities	To access medical services in (Colombian) hospitals, migrants must be admitted (they cannot receive outpatient care), which implies procedures within the institution's social work offices for costs to be covered by territorial entities. Once admitted, several migrants have reported being held by the institution's medical staff until hospital fees were collected. For those who leave the hospital with debt, they are threatened with refusal of further medical attention if they do not pay their fees on time. In Peru, 53% of participants mentioned situations of discrimination in the health system in Peru. Discriminatory attitudes toward migrants often come from medical staff.	We conclude that COVID-19 has exacerbated preexisting conditions of informality and health inequities affecting Venezuelan migrants in Colombia and Peru. These findings also provide guidance for specific policy recommendations. Given legal and financial obstacles, it is important to facilitate access to public health services irrespective of migratory status. Access should give special attention to COVID-19 care in the short and medium term but should focus on creating and strengthening systems of access in the long term. Regarding information asymmetry, it is critical to focus on both the 'demand' and 'supply' sides; national and municipal authorities should develop, promote, and consistently disseminate up-to-date information on access options all along the various points of friction in the bureaucratic itineraries of care (this includes hospital security staff, support personnel, secretarial staff, etc.), and must consider preferred digital platforms. Training sessions for public health staff on national migration law and information on access to services is necessary, as this would facilitate the overall usage of public health services amongst migrants and decrease discrimination practices by staff. Alternative purveyors of care should be approached as important resources in these tasks of information dissemination as they already effectively work as intermediaries between migrants and official health providers. Finally, and specifically for resource-constrained public sector agencies, policy should improve procedural and operational options to support and strengthen those alternative sources of care that can be effective and safe, such as telemedicine, especially as they are becoming active parts of the overall system in Colombian and Peruvian cities.

**Author, Year, Title**	**Findings**	**Conclusions / Recommendations**
Zihindula, G, 2017, Lived Experiences of Democratic Republic of Congo Refugees facing Medical Xenophobia in Durban, South Africa	Interviews conducted with 31 Congolese refugees revealed that medical xenophobia is often experienced almost at every encounter between a refugee and a health care worker. Findings in this study revealed that communication and documentation are the two main encounters that affect any other services, including denying services or providing poor services. Health care workers' attitudes, stereotyping, negligence, poor listening and cultural insensitivity were reported from the part of the providers. Under-diagnosis, underutilization of treatments and services, misdiagnosis and intervention misuse were the medical xenophobia's outcomes revealed by the study. An increased level of trauma and stress were also expressed by participants who reported medical xenophobia and discrimination.	In order to improve refugees' access to health care services, health care workers need to be educated about how to treat refugees seeking health care in their facilities.
